# Fe/N‐Doped Carbon with Nearly Ordered Mesopores and Tunable Particle Sizes as Model Catalysts for Quantitative Evaluation of Electrocatalytic Active Sites

**DOI:** 10.1002/advs.202519066

**Published:** 2026-02-04

**Authors:** Hongjuan Zhang, Yunqi Li, Jiacheng Zhao, Yue Liu, Xingtao Xu, Yusuke Yamauchi, Jing Tang, Min Zhou

**Affiliations:** ^1^ College of Physical Science and Technology Yangzhou University Yangzhou P. R. China; ^2^ State Key Laboratory of Petroleum Molecular and Process Engineering Shanghai Key Laboratory of Green Chemistry and Chemical Processes School of Chemistry and Molecular Engineering East China Normal University Shanghai P. R. China; ^3^ Beijing Key Laboratory of Bio‐inspired Energy Materials and Devices International Center for Energy and Environment School of Energy and Power Engineering Beihang University Beijing P. R. China; ^4^ Xin Feng Ming Group Huzhou Zhongshi Technology Co., Ltd. Huzhou P. R. China; ^5^ Marine Science and Technology College Zhejiang Ocean University Zhoushan P. R. China; ^6^ Department of Materials Process Engineering Graduate School of Engineering Nagoya University Nagoya Japan; ^7^ Australian Institute for Bioengineering and Nanotechnology (AIBN) The University of Queensland Brisbane Queensland Australia

**Keywords:** mesoporous carbon, model catalyst, ordered mesoporous MOF precursor, oxygen reduction reaction, tunable size

## Abstract

Synthesizing MOF‐derived carbons with not only tunable and uniform particle sizes but also ordered tubular mesoporous structures remains challenging. Moreover, the lack of precise morphological control makes it hard to clarify the relationships between structure and catalytic activity, limiting the rational design of MOF‐derived electrocatalysts with breakthrough performance. This study successfully prepares ordered mesoporous Fe/MOF‐545‐*x* rod‐shaped precursors with tunable lengths via a one‐step modulation approach. After direct carbonization, Fe/MNC‐*x* retains the rod‐shape, mesoporous structures, and well‐dispersed Fe‐N*_x_* active sites. Considering that the axis length is the most significant variable for the Fe/MNC‐*x* series, they are promising model electrocatalysts to reveal the relationship between the particle size and utilization efficiency of electrocatalytic active sites in ORR. The electrochemical result shows that Fe/MNC‐250 nm, which has the shortest length, displays a superior activity (*E*_1/2_ = 0.917 V in alkaline and *E*_1/2_ = 0.814 V in acidic electrolytes), due to the higher electrochemical surface area, lower charge transfer resistance, and a higher efficient active site density (1.27 ± 0.26 × 10^1^ ^9^ sites g^−^^1^) estimated by in situ nitrite stripping technique. The fuel cell assembled using Fe/MNC‐250 nm possesses an excellent power density of 521.16 mW cm^−^^2^. This work provides a simple strategy for regulating the particle sizes of ordered mesoporous MOF precursors and the derived carbon‐based electrocatalysts for high‐performance PEMFCs.

## Graphical abstract

This study is dedicated to the design and synthesis of MOF‐derived mesoporous Fe/N‐doped carbons as model catalysts, aiming to quantitatively evaluate the utilization of active sites in the ORR. Through modulation followed by pyrolysis, we successfully prepare size‐tunable Fe/MNC‐*x* with doped iron atoms and mesoporous structures. The smaller Fe/MNC‐250 nm exhibits superior ORR performance (excellent ECSA, high durability, half‐wave potentials of 0.917 V in alkaline and 0.814 V in acidic electrolytes, and 521.16 mW cm^−^^2^ power density in PEMFC tests). Moreover, as determined by in situ nitrite stripping, it has the highest *SD* value (1.27 ± 0.26 × 10^1^ ^9^ sites g^−^^1^), indicating abundant mesopores for fast mass transport and sufficient accessible surface Fe─N_4_ active sites for ORR. It addresses the critical challenges of synthesizing such carbons with tunable particle sizes and mesoporous structures, clarifying structure‐activity relationships. This work establishes a foundation for designing highly efficient MOF‐derived electrocatalysts for PEMFCs and other applications.

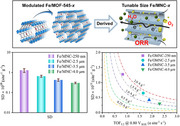

## 1 Introduction

Metal–organic framework (MOF) derived carbon materials have emerged as competitive electrocatalysts for oxygen reduction reaction (ORR) in proton exchange membrane fuel cells (PEMFCs) or metal‐air/O_2_ batteries, thanks to their high porosity, adjustable compositions, and structural features inherited from MOF precursors [1, 2, 3]. These features support efficient mass transport channels and rich active sites, showing potential to replace commercial Pt‐based catalysts [4, 5, 6]. However, traditional MOF‐derived carbons often have uncontrollable particle sizes, irregular micropores, and insufficiently exposed active sites [7, 8]. Oversized particles may trap active sites in internal carbon matrices, while undersized ones tend to agglomerate, reducing accessible surface areas and hindering mass transfer channels [9, 10]. Moreover, the lack of precise control over MOF precursor morphology makes it hard to clarify the relationships between structure and activity, limiting the rational design of high‐performance catalysts.

To tackle these issues, designing MOF precursors with tailored structures and converting them into carbon‐based catalysts with optimized properties has become crucial. Among various methods, modulator directed synthesis stands out as an innovative method to engineer MOF precursors [11, 12]. By adding modulators (e.g., organic acids or ligand fragment compounds) that compete with organic ligands for coordination with metal nodes, this approach precisely regulates MOF nucleation and growth [13, 14]. This competition not only enables the synthesis of MOFs with tunable particle sizes (from nanometers to micrometers) but also adjusts their porosity, crystallinity, and morphological uniformity [15, 16, 17]. Compared to conventional methods, the modulator strategy offers unique advantages in morphology control, such as avoiding complex synthesis conditions, ensuring consistency between experimental batches, and allowing systematic tuning of structural parameters without changing the basic MOF topology [18, 19]. This control factor is key to producing MOF derived carbons with predictable properties, laying the sense for studying modulated features dependent catalytic performance.

The modulator directed method has shown great potential in expanding MOF synthesis versatility. For example, by adjusting modulator types or concentrations, Kitagawa et al. reported that the crystal size of Zr‐MOF could be regulated by introducing modulators [20]. Behrens et al. utilized benzoic acid as a modulator to control the crystal size of UiO‐66 [21]. In addition, in our previous work, the mesoporous PCN‐224 with size‐tunable cubic shapes was prepared, and the derived nitrogen‐doped carbon retained structural features. Furthermore, incorporating iron single‐atom metal active sites into these MOF‐derived carbons effectively constructs high‐activity Fe─N*_x_* moieties [22]. These moieties are a critical factor in boosting ORR performance. This combination of modulated structure and active site engineering provides a solid platform for developing high‐performance electrocatalysts.

Herein, we attempted to apply the modulator strategy for regulating MOF‐545 [23], an ordered mesoporous porphyrin‐based MOF, to prepare iron‐incorporated MOF‐545‐*x* (Fe/MOF‐545‐*x*) with tunable particle sizes. Through high‐temperature carbonization, the iron single atom‐ and nitrogen‐doped mesoporous carbons (Fe/MNC‐*x*) with various particle sizes ranging from 250 nm to 4.0 µm were successfully obtained. We investigated the intrinsic relationship between particle size and electrochemical performance by comprehensive characterizations and systematic electrochemical evaluations. The effect of particle size on the utilization efficiency of Fe─N_4_ active sites was also carefully discussed. This work provides a versatile strategy for designing MOF‐derived electrocatalysts and deepens understanding of how structural parameters influence electrocatalytic behavior, promoting their application in fuel cells and other energy conversion devices.

## 2 Results and Discussion

### 2.1 Synthesis and Structural Characterizations

**FIGURE 1:**
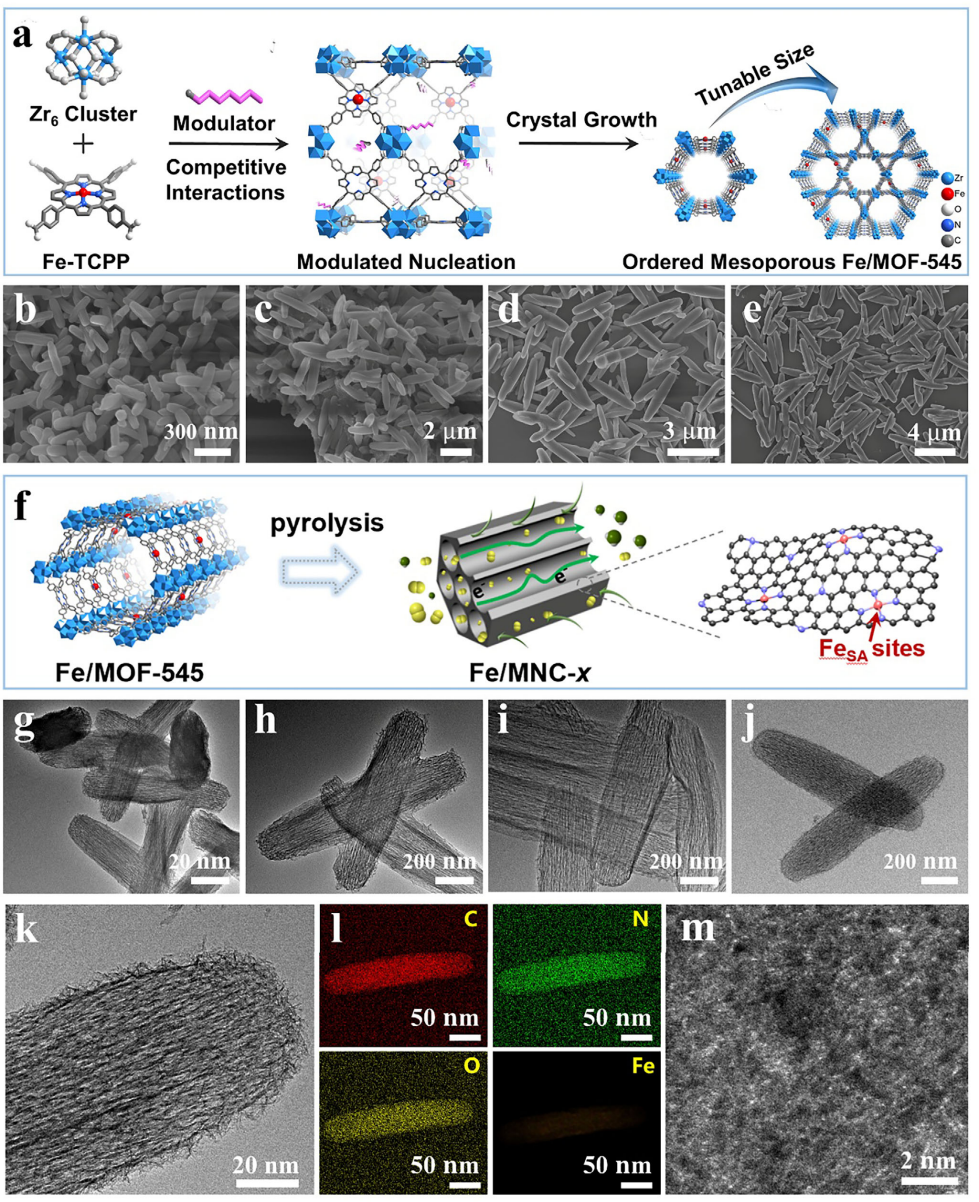
(a) Schematic illustration of the preparation process of ordered mesoporous Fe/MOF‐545. (b–e) SEM images of Fe/MOF‐545‐*x*. (f) Schematic illustration of the synthesis process of mesoporous Fe/MNC‐*x*. (g–j) TEM images of Fe/MNC‐*x*. (k) High‐magnification TEM image, (l) elemental mapping images, (m) CS‐TEM image of Fe/MNC‐250 nm.

The ordered mesoporous Fe/MOF‐545 with tunable length and sectional size was synthesized via a modulator approach (Figure 1a). In brief, the modulator was used as an additive to regulate the MOF crystal growth, and the size of the MOF crystallite and the number of defects within the bulk crystal can be systematically controlled by adjusting the concentration of modulators during synthesis. The Fe species are involved in the MOF precursor through adding Fe‐TCPP ligands. The metalloporphyrin ligands consist of isolated Fe species and are successfully implanted into the MOF‐545 lattices. To achieve tunable Fe/MOF‐545 precursors, the mass concentration of the modulator was precisely tailored by engineering TCPP/modulator molar ratios of y = 20/40, 15/45, 10/50, and 10/80. This rational design yielded four sets of crystals with uniform particle sizes spanning from the nanoscale to the microscale. As shown in Figure 1b–e, Fe/MOF‐545 with a length of ∼250 nm, 2.5 µm, 3.5 µm, 4.0 µm and a sectional size of ∼40, 200, 260, 300 nm were prepared, named as Fe/MOF‐545‐*x* (*x* = 250 nm, 2.5 µm, 3.5 µm, 4.0 µm) (Figure S1). The average particle size increases with elevated modulator concentration, indicating the modulator primarily exerts a dual role in promoting both nucleation and crystal growth during Fe/MOF‐545 formation. The Fe/MOF‐545‐*x* has significant advantages in the nanomaterials field, owing to its highly regular and mesoporous structures with ultrahigh surface area, along with abundant linker topology, and rich active sites.

As shown in Figure 1f, Fe/MNC‐*x* was obtained by pyrolysis of the Fe/MOF‐545‐*x* precursor. Comprehensive characterizations were carried out in order to understand the structural and compositional features of Fe/MNC‐*x*. Transmission electron microscopy (TEM) images in Figure 1g–j illustrated that Fe/MNC‐*x* kept a rod‐like morphology, nearly ordered mesoporous structure; and there were no visible iron agglomerates (Figure 1k). Furthermore, the energy‐dispersive X‐ray spectroscopy (EDS) elemental mapping revealed the homogeneous distribution of C, N, O, and Fe species over Fe/MNC‐250 nm (Figure 1l). The fine structure of Fe sites was identified by atomic‐resolution TEM. The bright spots of metal atoms were homogeneously dispersed on the carbon support (Figure 1m).

**FIGURE 2:**
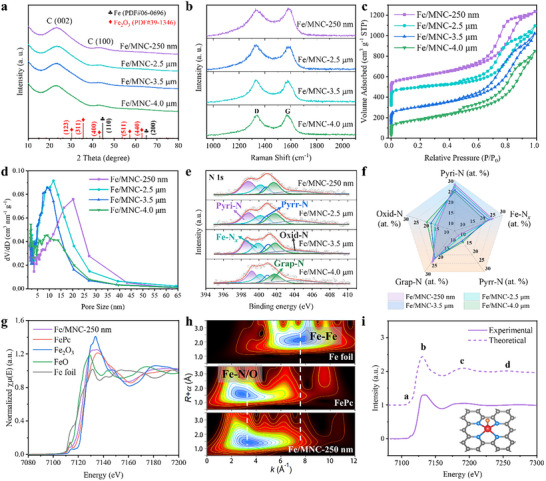
(a) XRD patterns and (b) Raman spectra of Fe/MNC‐*x*. (c) N_2_ adsorption–desorption isotherms and (d) pore size distributions of Fe/MNC‐*x*. (e) N1s spectra of Fe/MNC‐*x*. (f) Comparison of pyridinic N (Pyri‐N), pyrrolic N (Pyrr‐N), graphitic N (Grap‐N), Fe─N*_x_*, oxidized‐N (Oxid‐N). (g) Fe K‐edge XANES spectra for the Fe/MNC‐250 nm catalyst with reference samples (FeO, Fe_2_O_3_, FePc, and standard Fe foil). (h) WT‐EXAFS of the Fe/MNC‐250 nm, FePc, and Fe foil samples. (i) Comparison the K‐edge XANES between experimental spectrum and the theoretical spectrum. Structure diagrams of Fe active sites, colors represent Fe in red, N in blue, O in yellow and C in black.

The phase state of Fe/MNC‐*x* was investigated by X‐ray diffraction (XRD) (Figure 2a), all the samples exhibited two broad diffraction peaks at approximately two thetas of 23.5° and 44°, which were indexed to the (002) and (101) planes of graphite carbon (sp^2^ C) [24]. No characteristic crystal peaks of iron or metal oxides can be observed in the patterns of Fe/MNC‐*x*, excluding the existence of crystalline iron nanoparticles. Raman spectroscopy of Fe/MNC‐*x* showed that the intensity ratios of D band to G band (*I_D_/I_G_*) were 1.05, 1.03, 0.99, and 0.97 for Fe/MNC‐250 nm, Fe/MNC‐2.5 µm, Fe/MNC‐3.5 µm and Fe/MNC‐4.0 µm, respectively (Figure 2b), suggesting the similar degree of graphitization [25]. The N_2_ adsorption–desorption isotherms of Fe/MOF‐545‐*x* showed a typical type‐IV isotherms with specific surface areas of 603–676 m^2^ g^−^^1^ (Figure 2c) and the pore sizes ranged from ∼2.5 nm to 40 nm (Figure 2d). All Fe/MNC‐*x* displayed a steep N_2_ uptake at low relative pressure (*P/P_0_* = 0–0.015) and a well‐defined hysteresis loop at a higher relative pressure (*P/P_0_* = 0.45‐0.98), reflecting the coexistence of micropores and mesopores (Table S1). It's worth noting that the Fe/MNC‐*x* have expanded mesopores, when the particle length decreases from 4.0 µm to 250 nm (Figure 2d). The expanded mesopores are probably due to the accelerated collapse of the carbon matrix during calcination.

The X‐ray photoelectron spectroscopy (XPS) results revealed that Fe/MNC‐*x* exhibited similar element contents of C, O, N, and Fe (Figure S2a and Table S2). Notably, the nitrogen dopant in Fe/MNC‐*x* stems from the porphyrin ligand H_2_‐TCPP. The high‐resolution N 1s spectra and comparison of five types of N are presented in Figure 2e and Table S3. These results suggest that Fe/MNC‐*x* have similar N contents. In addition, the morphology of Fe/MNC‐*x* benefits the in situ generation of uniform nitrogen‐doping sites. The graphite N content of Fe/MNC‐*x* obtained at the same calcination temperature is almost equal, and the graphitic N surrounding the Fe─N*_x_* site dramatically impacts the spin state of Fe center, so its contribution to catalytic properties is similar. Especially, the Fe─N*_x_* bond with the binding energy of 399.2 eV [26], associated with the abundant atomically dispersed Fe─N*_x_* functional moieties in Fe/MNC‐*x*. The Fe‐TCPP as an organic ligand is perfectly coordinated in Fe/MOF‐545 precursor, so the Fe atom exists precisely as a single atom on the carbon support. Implying that Fe/MOF‐545 tends to form highly dispersive and completely accurate single atoms. As shown in Table S2, the Fe/MNC‐*x* (*x *= 250 nm, 2.5, 3.5, 4.0 µm) samples have surface Fe contents of 3.58, 3.51, 3.99, 3.94 at.%, respectively. These results indicate that the size change of catalysts did not substantially alter the Fe─N*_x_* moieties. Given the virtually identical active sites, these Fe/MNC‐*x* catalysts represent an ideal catalyst for exploring the impact of mesoporous morphology on the PEMFC performance.

The fine structure of the iron species was analyzed by X‐ray absorption spectroscopy. Figure 2g shows the Fe K‐edge X‐ray absorption near‐edge structure spectra (XANES) of Fe/MNC‐250 nm and reference samples. The adsorption threshold position of Fe/MNC‐250 nm located between FeO and Fe_2_O_3_ implies that the chemical valence of Fe in Fe/MNC‐250 nm is situated between +2 and +3 [27, 28]. The Fourier‐transformed extended X‐ray absorption fine structure (FT‐EXAFS) spectrum of Fe/MNC‐250 nm in Figure S3a shows a main peak at 1.5 Å in *R* space, close to the Fe─N peak of iron phthalocyanine (FePc) [29]. The comparison with Fe foil and oxide references shows that there is no apparent Fe─Fe bonds in Fe/MNC‐250 nm, suggesting that Fe species are atomically isolated by the surrounding N and C atoms. The above results are in line with the analysis of their wavelet transform (WT)‐EXAFS spectra. As shown in Figure 2h, the WT analysis of Fe/MNC‐250 nm shows only one higher intensity maximum at about 3.2 Å^−^^1^, which is close to the value of FePc (∼3.0 Å**^−^** ^1^). No typical Fe─Fe scattering shells are detected around 7.8 Å^−1^. This further confirms the isolated features of the metal Fe species in Fe/MNC‐250 nm. The FT‐EXAFS spectrum was well fitted by incorporating backscattering paths of Fe─N and Fe─O (Figure S3b). While the total average coordination number of Fe─N/O was approximately 5.0, the detailed fitting results include the partial coordination numbers of Fe─N and Fe─O; and the fitting goodness parameters (e.g., R‐factor, σ^2^), and corresponding error ranges are comprehensively presented in Table S4. The experimental spectrum showed a pre‐edge at ∼7115 eV, suggesting a deviation from perfect Fe─N_4_ square planarity (corresponding to a peak at ∼7118 eV) and the presence of an axial ligand [30]. The excellent agreement between experimental and theoretical spectra was obtained in Figure 2i, and a possible structure of the FeN_4_C_8_ moiety with one O_2_ molecules adsorbed (inset Figure 2i).

### 2.2 Electrochemical Performance in ORR

**FIGURE 3:**
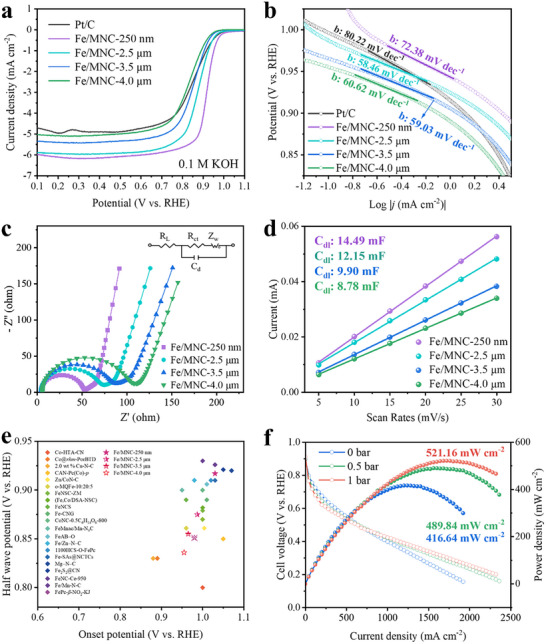
(a) LSV of Fe/MNC‐*x* and Pt/C at 1600 rpm with a scan rate of 10 mV s^−^^1^ in O_2_ saturated 0.1 M KOH. (b) Tafel plots. (c) EIS. (d) *C*_dl_ of Fe/MNC‐*x*. (e) Comparison of ORR activities between Fe/MNC‐250 nm and other reported examples. (f) Polarization and power density curves of catalysts. Test conditions: cathode loading of 3 mg cm^−2^.

Given that characterization results confirm the Fe/MNC‐*x* catalysts share highly similar structures and compositions, with nanoscale to microscale particle size as the sole critical variable, they serve as ideal model catalysts to exclusively probe the size effect on ORR performance. To identify the effective dispersion and rapid mass transfer characteristics of the catalytic sites on mesoporous pores, the oxygen reductive reaction (ORR) properties of Fe/MNC‐*x* catalysts were investigated in basic/acidic environments by rotating disk electrode [31, 32]. As seen from cyclic voltammetry curves (Figure S4), Fe/MNC‐250 nm exhibited the highest catalytic activity with a pronounced cathodic ORR peak at 0.875 V in the O_2_‐saturated 0.1 M KOH. As estimated from the linear sweep voltammetry (LSV) curves in Figure 3a, Fe/MNC‐250 nm shows the highest activity for ORR with an onset potential (*E*_onset_) of 1.028 V and a half‐wave potential (*E*_1/2_) of 0.917 V, which surpasses the *E*_1/2_ of 42, 62, 81 mV for Fe/MNC‐*x* (*x *= 2.5, 3.5, 4.0 µm) and is also more positive than that of commercial Pt/C (*E*_1/2_ = 0.850 V). The outstanding ORR activity and reaction kinetics during ORR are further evaluated by the Tafel slope in Figure 3b. The Tafel slopes of all the Fe/MNC‐*x* catalysts are significantly smaller than that of Pt/C (80.22 mV dec^−^^1^), indicating that the catalyst possesses significant advantages in reaction kinetics, the high intrinsic catalytic activity of single atom Fe sites is evident. Electrochemical impedance spectroscopy (EIS) studies were conducted (Figure 3c), and the results show that all the catalysts display a charge transfer process related to surface intermediates and porosity [33]. The ORR kinetics were probed by the Koutecky–Levich (K–L) equation (Figure S5a,b), and the good linear and parallel characteristics revealed a first‐order reaction. The electron transfer number (n) and H_2_O_2_ yield were further evaluated through rotating ring‐disk electrode [34], where the direct four‐electron transfer mediated ORR process was confirmed (the n approached 4 and the H_2_O_2_ yield was below 1.1%) (Figure S5c,d).

In order to find out the effective active site in Fe/MNC‐*x* corresponding to the catalytic activity, we explore its activity contribution from the electrochemical surface area (ECSA). The electric double‐layer capacitances (*C*_dl_) were measured by obtaining cyclic voltammetry (CV) curves in the non‐Faradaic potential range (Figure S6) [35]. Fe/MNC‐250 nm presents the largest *C*_dl_ of 14.49 mF (Figure 3d). The *C*_dl_ value is positively correlated with the ECSA [36, 37]. This result indicates that the ECSA is largely affected by the porous structure, and the mesoporous structure plays a critical role in the formation of the interphasebetween the electrode‐electrolyte interface. The stability of Fe/MNC‐250 nm for ORR was investigated by measuring the LSV after 5000 CV cycles. The results show an almost negligible decrease in the half‐wave potential, and only a slight decrease in the limiting current density (Figure S7). The electrocatalytic activity of Fe/MNC‐250 nm is compared with that of other reported non‐precious metal catalysts in Figure 3e, and details of the references are supplied in Table S5.

Encouraged by the excellent ORR performance of Fe/MNC‐250 nm in alkaline media, we further explore its ORR performance under more challenging acidic conditions (0.5 m H_2_SO_4_). In Figure S8a, the LSV curve of Fe/MNC‐250 nm shows considerable ORR activity but relatively lower half‐wave potential (0.814 V) and onset potential (*E*_onset_ = 0.10 V) than that of commercial Pt/C (*E*_onset_ = 0.101 V, *E*_1/2_ = 0.863 V), indicating that the Fe─N_4_ species as an active site exhibited the tremendous potential of ORR activity in the acidic electrolyte. To further elucidate the ORR process, the K–L plots and rotating ring‐disk electrode tests were carried out in Figure S8b–d. The good linear relationships suggest a first‐order reaction. In the potential window of 0.2–0.8 V, the H_2_O_2_ yields are below 8.6%, while the electron transfer number is above 3.8, confirming the typical four‐electron transfer mediated ORR process.

The excellent oxygen reduction catalytic performance of the catalyst is expected to have practical applications in hydrogen/oxygen energy storage and conversion devices, so the proton exchange membrane fuel cells (PEMFCs) were assembled to evaluate the performance of the catalyst in practical all‐battery applications [38, 39]. In Figure 3f and Figure S9, the excellent ORR activity in acidic media of Fe/MNC‐250 nm has been proved by the H_2_─O_2_ PEMFC measurements. The cathode with a catalyst loading of 3.0 mg cm^−2^ was tested under a range of H_2_/O_2_ backpressures (0, 0.5, and 1.0 bar). For Fe/MNC‐250 nm with rich mesoporous features, the peak power density reaches a considerable value of 521.16 mW cm^−^^2^ under 1.0 bar of pressure, and its impedance is low, clearly demonstrating the vital importance of the porous structure in Fe/MNC‐250 nm for the improvement of fuel cell performance. These results show that the catalytic performance of Fe/MNC‐250 nm is close to practical applications.

### 2.3 The Catalytic Mechanism Investigation for Fe/MNC‐*x*

**FIGURE 4:**
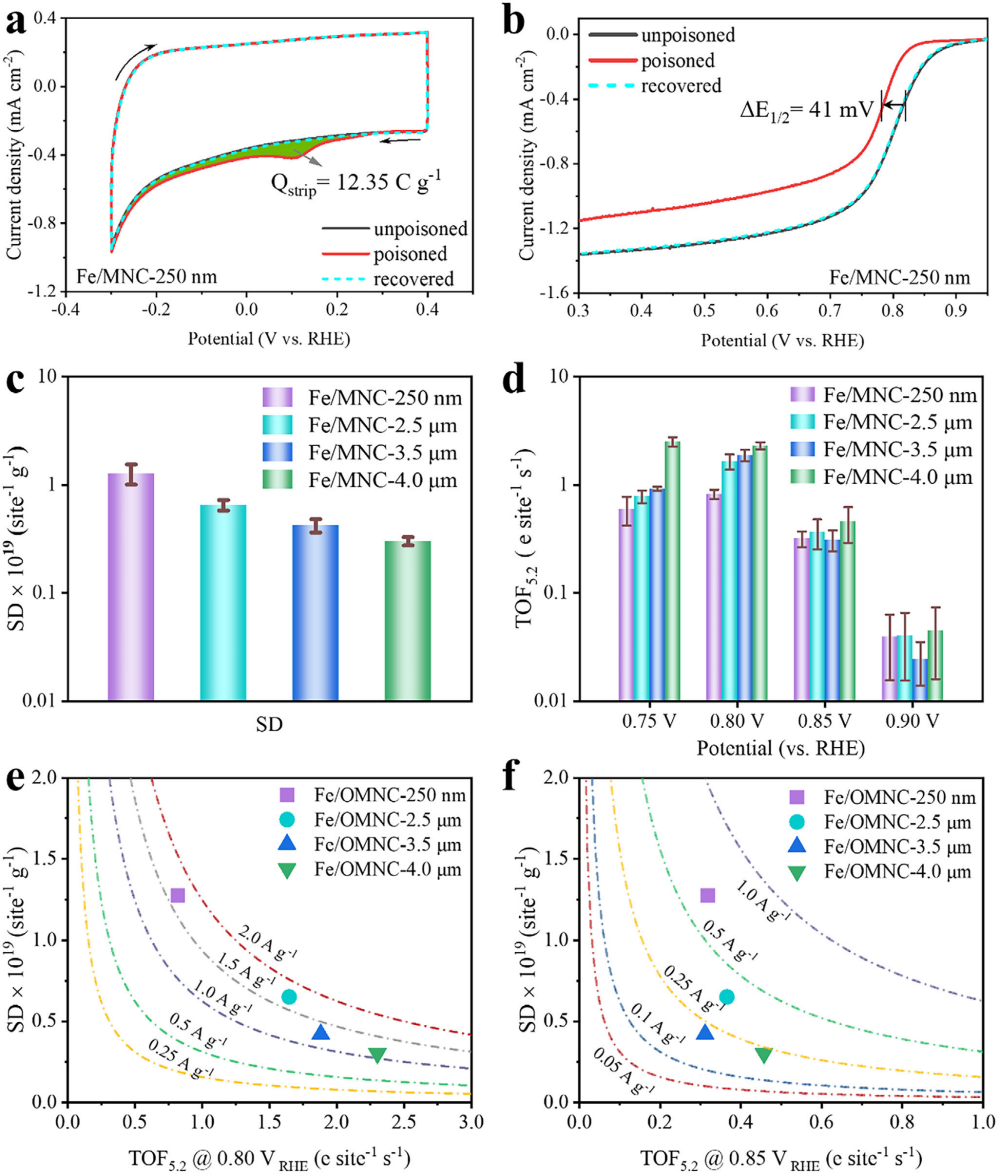
(a) CV and (b) LSV curves of the unpoisoned, nitrite‐poisoned, and recovered Fe/MNC‐250 nm electrodes in 0.5 m acetic acid buffer electrolyte. (c) SD values and (d) TOF at 0.80, 0.85, and 0.90 V of Fe/MNC‐*x* calculated from the nitrite stripping method in 0.5 m acetic acid buffer electrolyte. Isoactivity plots at (e) 0.80 V and (f) 0.85 V.

In order to figure out the influence of morphology on the effective numbers of Fe─N_4_ active sites, in situ nitrite stripping techniques on Fe/MNC‐*x* were conducted [40]. For the purpose of enhancing experimental reproducibility, a 0.5 m acetate buffer solution with a pH of 5.2 was utilized as the electrolyte. This optimized condition guarantees that nitrite reduction proceeds with high facility, while simultaneously ensuring the adequate stability of nitrite anions. It can be seen from the CV curves in Figure 4a, there is a discernible difference between the unpoisoned and poisoned Fe/MNC‐250 nm electrodes during a quite narrow potential range, which is the nitrite reductive stripping region. The reductive stripping charge is perfectly correlated with the poisoned Fe─N_4_ active sites on the electrode which has been pre‐exposed to nitrite solution. After performing stripping by CV, the poisoned electrode is completely recovered and shows an overlapped CV curve with the unpoisoned electrode. The total amount of stripping charge (*Q*_strip_) associated with the stripping peak of Fe/MNC‐250 nm is 12.35 C g^−^^1^ (inset of Figure 4a). The amount of stripped charge is directly correlated to the decrease and recovery level of the catalyst performance. As shown in Figure 4b, the ORR performance of the nitrite‐poisoned electrode is reduced significantly, displaying a negative shift of 41 mV for *E*_1/2_ on its poisoned state. After carrying out stripping by CV, the poisoned electrode is completely recovered and shows an overlapped LSV curve with the unpoisoned electrode. In situ nitrite stripping technique was also operated on the other three samples (Figure S10). The calculated *Q*_strip_ of Fe/MNC‐2.5 µm, Fe/MNC‐3.5 µm and Fe/MNC‐4.0 µm are 5.82, 4.32, and 2.26 C g^−^^1^, respectively, which are markedly lower than that of Fe/MNC‐250 nm (12.35 C g^−^^1^). The various values of *Q*_strip_ indicate that the number of effective Fe─N_4_ sites is negatively correlated with particle size length.

The number of Fe─N_4_ sites was calculated based on the nitrite stripping method. It is assumed that the Fe─N_4_ active sites interacted with nitrite in a manner analogous to the iron heme complex, with nitrite being converted into a nitrite ligand during the poisoning process. By analyzing the stripped nitrite intermediate, the value of *Q*_strip_ can be determined, and the quantity of active sites is equivalent to the number of stripped nitrite ligands. The active sites density (SD) can be calculated from the number of Fe─N_4_ sites normalized to the mass. As compared in Figure 4c, the SD value has the same trend as *Q*_strip_. Fe/MNC‐250 nm exhibits the highest SD value of 1.27 ± 0.26 × 10^19^ sites g^−^^1^, which is much higher than those of Fe/MNC‐2.5 µm (0.65 ± 0.07 × 10^19^ sites g^−1^), Fe/MNC‐3.5 µm (0.42 ± 0.05 × 10^19^ sites g^−^^1^), and Fe/MNC‐4.0 µm (0.30 ± 0.02 × 10^19^ sites g^−^^1^). TOF_5.2_ values (the subscript represents the pH of electrolyte in which turnover frequency (TOF) is estimated) were assessed at four potentials and summarized in Figure 4d. Fe/MNC‐*x* shows a volcanic trend in TOF_5.2_ along with the increased potentials with a maximum value at 0.8 V. The TOF_5.2_ values of Fe/MNC‐250 nm, Fe/MNC‐2.5 µm, Fe/MNC‐3.5 µm, and Fe/MNC‐4.0 µm at 0.80 V are 0.82 ± 0.07, 1.64 ± 0.26, 1.88 ± 0.22, and 2.30 ± 0.17 e site^−^^1^ s**^−^** ^1^, respectively, enhancing almost three times when the length increased from 250 nm to 4 µm. The number of accessible active sites in the catalysts is closely related to their support microenvironment. Owing to the size‐dependent mesoporous mass transfer effects, our catalysts with radial dimensions ranging from hundreds of nanometers to hundreds of micrometers exhibit distinct mass transfer behaviors for reaction media. Consequently, significant discrepancies are observed in the experimentally measured TOF values. Isoactivity plots of Fe/MNC‐*x* catalysts were drawn using the SD and TOF_5.2_ values at 0.80 and 0.85 V. As shown in Figure 4e,f, the Fe/MNC‐250 nm possesses an exceptionally high SD, with an improvement ranging between 2‐ and 4‐times when compared with those of the other three catalysts. Among the four catalysts, Fe/MNC‐250 nm combines the highest SD with a relatively high TOF, which leads to high ORR catalytic performance. In practical processes, besides the dispersion of single‐atom Fe, the support microenvironment is also critical. Our rod‐like catalysts feature radial dimensions from hundreds of nanometers to micrometers, rendering the internal single‐atom Fe sites barely accessible to electrolytes, O_2_, and other reactants. Larger‐sized catalysts suffer from sluggish mesoporous mass transfer, including infiltration of reactants and desorption of products. Thus, 100% atomic utilization is unattainable. Despite comparable Fe loading across different sizes, the number of electrochemically accessible active sites varies significantly due to size‐dependent mesoporous mass transfer limitations. The above results clearly suggest that the smaller size of catalysts plays a key role not only in improving the SD but also in balancing the TOF of the electrochemically accessible Fe─N_4_ sites (Figure S11).

**FIGURE 5:**
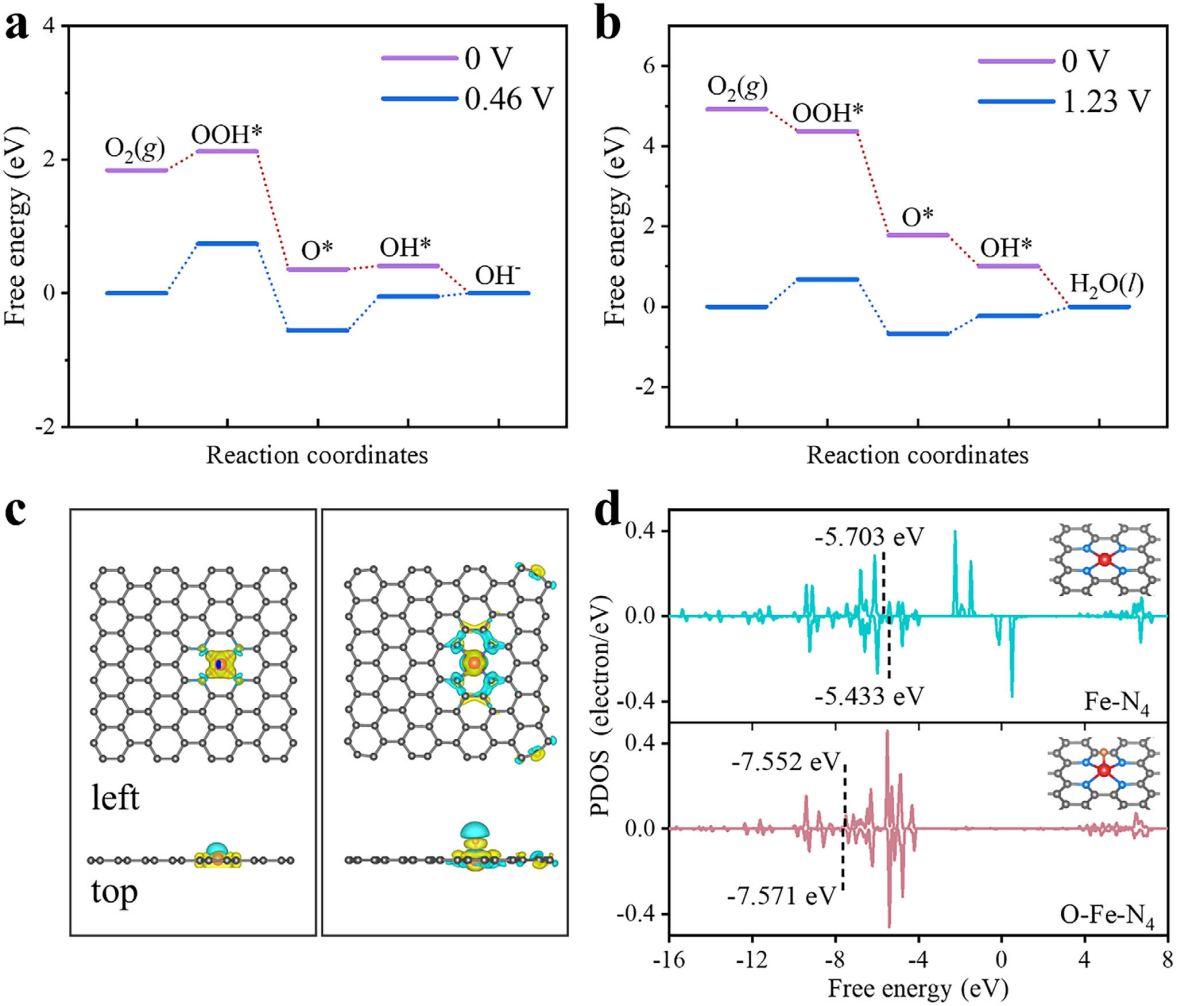
Free energy diagrams for the ORR process on the O─Fe─N_4_ site in (a) alkaline and (b) acidic conditions. (c) Differences in the charge densities (cyan and yellow regions represent increases and decreases in the charge density, respectively). (d) DOS of Fe 3d for the Fe─N_4_ and O─Fe─N_4_ sites.

Density functional theory calculations were performed to elucidate the origin of catalytic activity of the Fe/MNC‐*x* catalysts [41, 42]. Based on the previous discussion of structural and compositional characterization, O─Fe─N_4_ was constructed as the active site‘s structure model as shown in Figure S12. Considering that the alkaline and acidic electrolytes have an impact on the electrocatalytic reaction, the solvent effect was calculated at different equilibrium potentials to investigate the catalytic mechanism. The optimized structures of the ORR adsorbed intermediates (OOH^*^, O^*^, and OH^*^) at active sites are shown in Figure S13. The free‐energy diagrams are presented in alkaline media at U = 0.46 V (Figure 5a), the free energy is 1.30 eV for the two rate‐determining steps (OOH^*^ and OH^*^), and the lower energy barrier indicates rapid reaction kinetics. In acidic media U = 1.23 V (Figure 5b), the OOH^*^ and OH^*^ electron‐transfer steps are the sluggish rate‐determining steps, the free energy is calculated to be 1.36 eV. The O─Fe─N_4_ site at both equilibrium potentials has lower formation energies, which is thermodynamically favorable. The corresponding theoretical calculation parameters are shown in Tables S6–S8.

The charge redistribution of model structures of the catalyst's active sites Fe─N_4_ and O─Fe─N_4_ is clear (Figure 5c), revealing that the electrons transfer from iron to nitrogen atoms, resulting in a decrease in the charge densities of Fe. This is beneficial in weakening the adsorption energy of the intermediates on the active site during the ORR. The adsorption energies of reaction intermediates determine the activity of the oxygen reduction catalytic reaction. Thus, when a catalyst exhibits high ORR activity, the adsorption energy of the intermediates should be neither too weak to limit the subsequent reactions nor too strong to hinder the desorption of the intermediates. In Figure 5d, the density of states (DOS) of Fe 3d for Fe─N_4_ and O─Fe─N_4_ sites were calculated to evaluate the binding strength of the active sites to the reaction intermediates [43]. The d‐band center of O─Fe─N_4_ is significantly shifted downward compared to Fe─N_4_, and the lower DOS is beneficial for the desorption of intermediates, which can effectively reduce the reaction energy barrier and play a crucial role in the improvement of the ORR performance of Fe/MNC‐*x*.

## 3 Conclusions

In this work, we have successfully prepared the Fe/MNC‐*x* with iron atomic dispersion via an in situ modulated method followed by pyrolysis treatment. The obtained Fe/MNC‐*x* retained the mesoporous structures, and a wide size rang from micron to nanometer. The ORR performance of Fe/MNC‐*x* shows obvious regular changes when the size decreases. The smaller size Fe/MNC‐250 nm presents superior ORR performance along with a four‐electron reaction mechanism, excellent ECSA and high durability. The half‐wave potential of Fe/MNC‐250 nm in alkaline and acidic electrolytes was 0.917 and 0.814 V vs. RHE, respectively. Moreover, in H_2_─O_2_ PEMFC tests, a maximum power density of 521.16 mW cm^−2^ and lower impedance were obtained. The Fe/MNC‐250 nm had the highest SD value of 1.27 ± 0.26 × 10^19^ sites g^−^^1^, as determined by in situ nitrite stripping method. The results illustrated that the Fe/MNC‐250 nm would possess abundant mesopores for fast mass transport and enough accessible surface Fe─N_4_ active sites for ORR. This work breaks the size limitation of conventional MOF‐derived carbon to synthesize a series of tunable size MOF‐derived porous single atom metal doping carbon‐based catalysts, setting the scene for the design of highly efficient electrocatalysts for the PEMFCs or other electrocatalysis and energy storage applications.

## Conflicts of Interest

The authors declare no conflicts of interest.

## Supporting information




**Supporting File**: advs74062‐sup‐0001‐SuppMat.docx.

## Data Availability

The data that support the findings of this study are available from the corresponding author upon reasonable request.
