# Supplementary material for: Fe/N‐Doped Carbon with Nearly Ordered Mesopores and Tunable Particle Sizes as Model Catalysts for Quantitative Evaluation of Electrocatalytic Active Sites
Source: Adv Sci (Weinh). 2026 Feb 4;13(19):e19066. doi: 10.1002/advs.202519066 (PMC13045417; doi:10.1002/advs.202519066)
Supplement: Supplementary file 1 — Supporting File: advs74062‐sup‐0001‐SuppMat.docx. [file ADVS-13-e19066-s001.docx]

**Supporting Information**

**Fe/N-Doped Carbon with Nearly Ordered Mesopores and Tunable Sizes as Model Catalysts for Quantitative Evaluation of Electrocatalytic Active Sites**

Hongjuan Zhang^a,b^, Yunqi Li^c*^, Jiacheng Zhao^d^, Yue Liu^b^, Xingtao Xu^e^, Yusuke Yamauchi^f,g*^, Jing Tang^b*^, Min Zhou^a*^

^a^ College of Physical Science and Technology, Yangzhou University, Yangzhou 225002, China

^b^ State Key Laboratory of Petroleum Molecular and Process Engineering, Shanghai Key Laboratory of Green Chemistry and Chemical Processes, School of Chemistry and Molecular Engineering, East China Normal University, Shanghai 200062, China

^c^ Beijing Key Laboratory of Bio-inspired Energy Materials and Devices, International Center for Energy and Environment, School of Energy and Power Engineering, Beihang University; Beijing 100191, PR China

^d^ Xin Feng Ming Group Huzhou Zhongshi Technology Co., Ltd., Huzhou 313099, China

^e^ Marine Science and Technology College, Zhejiang Ocean University, 316022 Zhoushan, China

^f^ Department of Materials Process Engineering, Graduate School of Engineering, Nagoya University, Nagoya 464-8601, Japan

^g^ Australian Institute for Bioengineering and Nanotechnology (AIBN) and School of Chemical Engineering, The University of Queensland, Brisbane, Queensland 4000, Australia

E-mails: yunqi_li@buaa.edu.cn (Y. Li); y.yamauchi@nagoya-u.jp (Y. Yamauchi); jingtang@chem.ecnu.edu.cn (J. Tang); [minzhou@yzu.edu.cn](mailto:minzhou@yzu.edu.cn) (M. Zhou).

**Experimental Section**

**1. Chemicals****.** Zirconyl chloride octahydrate (ZrOCl2·8H2O, Aladdin Industrial Corporation, 98%), iron(II) chloride tetrahydrate (FeCl_2_·4H_2_O, Shanghai Macklin Biochemical Co., Ltd. 99.95%), 1H-pyrrole (Shanghai Acmec Biochemical Co., Ltd, 98%), L-cysteine methyl ester hydrochloride (Bide Pharmatech Co., Ltd. 98%), *N,N*-dimethyl formamide (DMF, 99.5%), methanol (CH_3_OH, 99.5%), anhydrous tetrahydrofuran (THF, 99.5%),ethyl acetate (CH3COOEt, 98.5%, CP), carbon tetrachloride (CCl_4_, 99.5%), dichloromethane (CH_2_Cl_2_, 99.5%), ethanol (CH3CH2OH, 99.7%), propanoic acid (C2H5COOH, 99%), trifluoroacetic acid (CF_3_COOH, 99.5%), methyl 4-formylbenzoate (98%), potassium hydroxide pellets (KOH, 99%), sulfuric acid (H_2_SO_4_, 98%), sodium hydroxide pellets (NaOH, 96%), hydrofluoric acid (HF, 40%) and hydrogen chloride (HCl, 36~38%) were all obtained from Sinopharm Chemical Reagent Co., Ltd. Ultrapure water was purchased from Hangzhou Wahaha Group Co., Ltd. Nafion D-520 dispersion (5% w/w in water and 1-propanol), and Pt/C (20 wt.%) were purchased from Alfa Aesar Chemical Reagent. All the chemicals used in this work were of analytical grade and were used without further purification.

**2. Synthesis of porphyrin ligand H_2_-TCPP (tetrakis (4-carboxyphenyl) porphyrin).** The porphyrin ligand was prepared according to the literature ^[1]^. In a typical synthesis, 200 mL round-bottomed flask with 3.0 g 1H-pyrrole and 6.9 g methyl 4-formylbenzoate. Then, 100 mL of propanoic acid was added to the above chemicals. The mixture was then refluxed at 140℃ under magnetic stirring for 12 h in darkness. After the mixture was cooled down, the products were washed in the sequence of ethanol, ethyl acetate and THF. The obtained purple products were then dried at 60°C under vacuum, the 5,10,15,20-tetrakis (4-methoxycarbonylphenyl) porphyrin was obtained. 200 mL round-bottomed flask with 0.75 g purple products and 25 mL CH_3_OH. Subsequently, 25 mL of an aqueous solution containing 2.65 g of KOH was added quickly into the flask under magnetic stirring. Then, the mixture was refluxed at 85℃ for 12 h. After the mixture cooled down. Cautiously add, dropwise, 1M HCl, allowing the solution pH to subside and again adding, accompanied by the formation of precipitate until pH reached 3. Finally, the obtained products were collected by suction-filtration, washed with water and dried under vacuum at 60℃, the porphyrin ligand tetrakis (4-carboxyphenyl) porphyrin (H2-TCPP) was obtained.

**3. Synthesis of metalloporphyrin ligand Fe-TCPP ([5,10,15,20-tetrakis (4-carboxyphenyl) porphyrinato]-Fe (III) chloride).** The raw material was similar the process used to prepare H2-TCPP, the purple products were obtained. 0.85 g purple products and 2.5 g FeCl_2_·4H_2_O in 100 mL DMF were refluxed at 160℃ under magnetic stirring for 6 h. Then, 100 mL water was added the cooled mixture, the products were collected by suction-filtration and washed with a little water. The obtained precipitate was dissolved in CHCl_3_, the organic solvent was extracted using 1 M HCl and water three times, respectively. Then, the organic layer was evaporated to afford dark brown crystals [5,10,15,20-tetrakis (4-methoxycarbonylphenyl) porphyrinato]-Fe (III) chloride. 200 mL round-bottomed flask with 0.75 g dark brown crystals and 25 mL CH_3_OH. Subsequently, 25 mL of an aqueous solution containing 2.65 g of KOH was added quickly into the flask under magnetic stirring, the mixture was refluxed at 85℃ for 12 h. After cooling down to room temperature, the solvent of mixture was evaporated. Then, additional H_2_O was added to completely dissolved the solid, the obtained homogeneous solution was acidified with 1 M HCl under stirring until no new formation of precipitate. The brown product was collected by filtration, washed with H_2_O and dried under vacuum at 65℃, the porphyrin ligand [5,10,15,20-tetrakis (4-carboxyphenyl) porphyrinato]-Fe (III) chloride (Fe-TCPP) was obtained.

**4. Preparation of Fe/ordered mesoporous nitrogen-doped carbon (Fe/MNC).** 25 mL round-bottomed flask with 108 mg ZrOCl2·8H2O, 10 mg Fe-TCPP, 10 mg H_2_-TCPP and 40 mg modulator (L-cysteine methyl ester hydrochloride). Then, 10 mL of DMF and 450 μL CF_3_COOH was added to the above chemicals, the mixture was then refluxed at 120℃ under magnetic stirring for 18 h. After the mixture cooled down. The obtained rod-like Fe-MOF-545-250 nm was collected by filtration, washed with DMF and acetone dried under vacuum at 65℃. To prepare rod-like Fe-MOF-545 with different section size and length, the amount of modulator added into the precursor solution was adjusted. The Fe-MOF-545-*x* (*x* represent the length of the rod-like Fe-MOF-545, *x* = 250 nm, 2.5 μm, 3.5 μm, 4.0 μm) were synthesized with different mass ratios of TCPP/modulator -*y* (*y* = 20/40, 15/45, 10/50, 10/80). Finally, the Fe/MNC-*x* was synthesized by directly calcining the Fe-MOF-545-*x* at a proper heating procedure which from room temperature to target temperature with a heating rate of 5℃ min^-1^ and kept at the target temperature (900, 1000, and 1100℃) for 2 h under N_2_ atmosphere. The final calcination temperatures were 1000℃. After cool to room temperature, the obtained sample was immersed into a solution of HF (5 wt.%) at 80℃ for 8 h to remove the ZrO_2_, then the clearly washed black sample was calcined again at target temperature, and the Fe/MNC-*x* (*x* = 250 nm, 2.5, 3.5, 4.0 μm) were obtained.

**5. Characterizations.**

The morphology of samples was obtained on a field emission scanning electron microscope (FESEM, Zeiss Gemini450) with an accelerating voltage of 5.0 kV. Transmission electron microscopy (TEM), scanning transmission electron microscopy (STEM) images, energy-dispersive X-ray spectroscopy (EDS), and elemental mapping analysis were performed using a JEOL JEM-2100F operated at 200 kV. Aberration-corrected transmission electron microscopy (CS-TEM, JEM-ARM300/JEOL) operated at 300 kV was used to characterize the fine structure. A rigaku rint 2000 X-ray diffractometer measured wide-angle X-ray diffraction (XRD) patterns with monochromatic Cu K*_α_* radiation (40 kV, 40 mA) at a scanning rate of 5° min^-1^. Brunauer-Emmett-Teller (BET) surface area and pore size measurements were performed with N_2_ adsorption/desorption isotherms at 77.5 K on a micromeritics ASAP 2020 V4.01 H instrument. The specific surface area was evaluated by the multipoint BET method at a relative pressure from 0.03 to 0.6 based on the adsorption data. X-ray photoelectronic spectroscopy (XPS) spectra were collected on a Thermo Scientific K-Alpha instrument with an Al K*_α_* radiation (15 kV, 6 mA). Fe K-edge X-ray absorption spectra (XAFS) were performed with Si (111) crystal monochromators at the BL14W Beam line at the Shanghai Synchrotron Radiation Facility (SSRF) (Shanghai, China). Before the analysis at the beamline, samples were placed into aluminum sample holders and sealed using Kapton tape film. The XAFS spectra were recorded at room temperature using a 4-channel Silicon Drift Detector (SDD) Bruker 5040. Fe K-edge extended X-ray absorption fine structure (EXAFS) spectra were recorded in transmission/fluorescence mode. The XAFS spectra of these standard samples were recorded in transmission mode. The spectra were processed and analyzed by the software codes Athena.

**6. Electrochemical analysis in three-electrode system.** The oxygen reductive reaction (ORR) performances were measured on an electrochemical workstation (CHI 760E, Shanghai Chenhua, China; and ALS rotating ring-disk electrode (RRDE) instrument, Japan) by constructing a three-electrode system. Platinum and saturated calomel electrodes (SCE) were selected as the counter electrode and reference electrode, respectively. The working electrode was prepared by dropping 5 µL of ink of catalyst on the polished RRDE glass carbon electrode with a diameter of 4 mm and dried under an infrared lamp. The catalyst loading amount was 0.2 mg cm^-1^ in alkaline electrolyte (0.1 M KOH)，and the catalyst loading amount was 0.6 mg cm^-1^ in acid electrolyte (0.5 M H_2_SO_4_). The ink was prepared by dispersing 5 mg of catalyst in 1 mL of mixed solvent, containing 50 µL of 5.0 wt.% Nafion solution, and water/isopropanol (v/v 1:2). The ink is well prepared by ultrasonication for 30 min. As for electrochemical measurement, the cyclic voltammetry (CV) tests of the catalyst under N_2_- and O_2_-saturated electrolyte were performed at 50 mV s^-1^, the polarization curves for ORR were performed in 0.1 M KOH or 0.5 M H_2_SO_4_ saturated with O_2_/N_2_ with a scan rate of 10 mV s^-1^ and rotating speed of 1600 rpm. The polarization curves for ORR were carried out by the rotating ring-disk electrode (RRDE) technique, and the ring electrode potential was set to 1.5 V vs. reversible hydrogen electrode (RHE). The rotating speeds of linear sweep voltammetry (LSV) measurements for working electrode were 400, 625, 900, 1225, 1600, 2025, and 2500 rpm. Cyclic voltammogram measurements of catalyst were performed in the non-Faradaic region from 1 to 1.1 V vs. RHE with various scan rates (5, 10, 15, 20, 25, and 30 mV s^-1^) under O_2_ atmosphere to obtain the double layer capacitance (C_dl_, mF cm^-2^). The slope of the double-layer charging current versus the scan rate plot corresponds to the 2C_dl_.

The electrochemical surface area (ECSA) of the electrode was calculated by the following equation:

ECSA = C_dl_/C_s_ (1)

Where C_s_ refers to the double layer capacitance/specific capacitance of the electrode. The C_s_ for a flat surface is generally found to be in the range of 20-60 μf cm^-2^. Herein, we used C_s_ value of 40 μf cm^-2^. Note that the ECSA calculated from the H_upd_ is suitable for the precious metal catalysts, but is not reasonable for carbon-based catalysts for the corresponding ECSA usually includes the area of carbon which is not the intrinsic active sites. Herein, the C_dl_ is used to estimate the ECSA.

Electrochemical impedance spectroscopy (EIS) measurements were carried out by applying an amplitude with 10 mV in a frequency range from 100 kHz to 0.1 Hz under open circuit voltage in 0.1 M KOH and 0.5 M H_2_SO_4_ electrolyte. All potential values were calibrated to the E_RHE_ based on the Nernst equation of E_RHE_ = E_SCE_ + 0.2415 + 0.0591 * pH. The ORR results were presented after subtractions of the currents measured in N_2_-saturated 0.1 M KOH or 0.5 M H_2_SO_4_ solution to remove capacitive currents. All current densities were calculated based on the geometric area of the rotating disk electrode after correcting.

For kinetic analysis by RRDE measurements, the electron transfer number (*n*) and H_2_O_2_ yield (*% H_2_O_2_*) were calculated from the following equations:

$n=4\times\frac{I_{D}}{I_{D}+I_{R}/N}$ (2)

$\% H_{2}O_{2}=2\times\frac{I_{R}/N}{I_{D}+I_{R}/N}\times100\%$ (3)

Where *I_D_* is the disk current; *I_R_* is the ring current, and *N* is the collection efficiency of the Pt ring on the RRDE electrode (0.37 in this work).

Another kinetic analysis by RDE measurements, current density (*J_K_*), and electron transfer number (*n*) was calculated from the Koutecky-Levich equation:

$J^{-1}={J_{K}}^{-1}+{J_{L}}^{-1}={J_{K}}^{-1}+{(B\omega^{0.5})}^{-1}$ (4)

$B=0.2nF{(D_{O_{2}})}^{2/3}\tau^{{-1}/6}C_{O_{2}}$ (5)

Where, *J* is the measured current density; *J_K_* and *J_L_* represent the kinetic and diffusion limiting current densities; *ω* means the rotating rate of the electrode; the constant 0.2 is used to determine *B* when the unit of rotation speed is rpm; *n* indicates the overall electron transfer number in O_2_ reduction; *F* is the Faraday constant (96485 C mol^-1^); $D_{O_{2}}$stands for the diffusion coefficient of O_2_ in the electrolyte at 20℃ (1.9$\times$10^-5^ cm^2^ s^-1^); $C_{O_{2}}$denotes the saturated concentration of O_2_ (1.2$\times$10^-6^ mol cm^-3^) in the aqueous system; *τ* represents the kinematic viscosity of the electrolyte solution (0.01 cm^2^ s^-1^). The durability of catalyst was also evaluated by measuring the LSV after 5000 continuous cyclic voltammetry cycles between 0.4 and 1.0 V (vs. RHE) at a scan rate of 50 mV s^-1^ under O_2_ atmosphere.

**7. In-situ** **nitrite stripping experiments.** The Fe-N₄ active sites of the catalysts were determined in situ via the nitrite stripping technique, and the results were used to quantify the Fe-N_4_ active site density (SD) and turnover frequencies (TOF). A catalyst loading of 0.2 mg cm^-2^ was chosen as loading in all experiments. All the nitrite stripping measurements were performed in a 0.5 M sodium acetate buffer as electrolyte (pH 5.2) by a three-electrode system. The experimental process is as follows:

**(1) Catalyst stabilization steps.** First, perform 20 CV cycles tests at a scan rate of 100 mV s^-1^, then conduct 10 CV cycles tests at a scan rate of 10 mV s^-1^ in nitrogen-saturated electrolyte within the potential window of 1.05-0.4 V. Next, carry out 3 CV cycles tests at a scan rate of 5 mV s^-1^ in an oxygen-saturated electrolyte within the potential window of 1.05-0.4 V. Repeat above steps twice. Afterwards, perform LSV tests in an oxygen-saturated electrolyte within the potential window of 1.0-0.3 V at a scan rate of 5 mV s^-1^. Conduct 6 CV cycles in nitrogen-saturated electrolyte at a scan rate of 10 mV s^-1^ within the potential window of 1.0-0.3 V. Additionally, record CV curves in a nitrogen-saturated electrolyte at a scan rate of 10 mV s^-1^ within the potential window of 0.4-0.3 V.

**(2)** **Catalyst poisoning steps.** The working electrode was immersed in a 125 mM NaNO₂ solution and maintained at open-circuit potential for 5 minutes with a rotation speed of 300 rpm. Then the working electrode immerses in deionized water at a rotation speed of 300 rpm for 1 minute and remove the surface excess NaNO_2_ on the working electrode. Perform LSV tests in an oxygen-saturated electrolyte within a potential window of 1.0-0.3 V at a scan rate of 5 mV s⁻¹, so as to record the oxygen reduction catalytic performance of the poisoned catalyst. Conduct CV tests in a nitrogen-saturated electrolyte at a scan rate of 10 mV s⁻¹ within a potential range of 1.0-0.3 V.

**(3) Catalyst unpoisoned steps.** In a nitrogen-saturated solution, CV tests were performed at a scan rate of 10 mV s⁻¹ within the potential range of 0.4-0.3 V to remove NO₂⁻ adsorbed on the active sites, this process was repeated until stability was achieved. Subsequently, in an oxygen-saturated electrolyte, LSV tests were conducted within the potential range of 1.0-0.3 V at a scan rate of 5 mV s⁻¹, and the oxygen reduction catalytic performance of the recovered catalyst was recorded.

The number of adsorbed NO₂⁻ species can be calculated by measuring the charge quantities before and after catalyst poisoning, thus enabling the determination of the number of active sites. The calculation formulas are presented below:

$Q_{strip (C g^{-1})}= \frac{\int i\mathrm{du}_{{(mA cm}^{-1} V)}}{v_{\left( {V s}^{-1} \right)} m_{({mg cm}^{-2})}}$ (6)

$\mathrm{MSD}_{\left( {mol site g}^{-1} \right)} = \frac{Q_{strip (C g^{-1})}}{n_{\mathrm{strip}} F_{(C \mathrm{mol}^{-1})}}$ (7)

$SD= MSD*N_{A}$ (8)

$\mathrm{TOF}_{(@0.8 V vs. RHE)(s^{-1})} = \frac{{\Delta i}_{k (A g^{-1})}}{F_{(C \mathrm{mol}^{-1})} \mathrm{MSD}_{({mol g}^{-1})}}$ (9)

In the above formulas: Q_strip_ denotes the nitrite reduction charge calculated from CV curves; I and u represent the Faraday current and applied potential for nitrite reduction, respectively; v and m denote the scan rate and catalyst loading on the electrode, respectively; n_strip_ is the number of electrons transferred during nitrite reduction (where *n*=5); F is the Faraday constant; MSD refers to the number of active sites per unit mass of the catalyst; Δi_k_ is the kinetic current difference of the catalyst at 0.8 V; The turnover frequency (TOF) of active sites is determined from the kinetic current on nitrite poisoning of the catalyst’s ORR.

**8. A ssemble of proton exchange membrane fuel cells (PEMFCs).** The Fe/MNC-250 nm catalyst (~10 mg) was mixed with Nafion alcohol solution (5 wt%, Aldrich), DI water (200 mg) and isopropanol (400 mg) to prepare the catalyst ink. The Nafion-to-catalyst ratio was 1.5. The ink was subjected to sonication for 10 min and stirring for 1 h to make a uniform suspension. The well-dispersed ink was brushed on a piece of carbon paper (5 cm^2^, GDS2240, Ballard), followed by drying in vacuum at 80°C for 2 h. As for anode, Pt/C (20 wt% of Pt, BASF) was used with a loading of ~0.5_mg_ Pt cm^-2^. The prepared cathode and anode were pressed onto the two sides of a Nafion 211 membrane (DuPont) at 130°C for 90 s under pressure of 1.5 MPa to obtain the MEA. The performance of MEA was measured by a fuel cell test station (Scribner 850e) with UHP-grade H_2_ and O_2_ at 80°C, 100% RH. The flow rates were 0.3l min^-1^ for H_2_ and 0.4l min^-1^ for O_2_. Polarization curves were recorded by scanning the current density with an increment of 20 mA cm^-2^ and the system is allowed to equilibrate by 3 s at each current step before a data point is recorded. EIS measurements were conducted at a constant current density of 1.5 A cm^-2^, in a frequency range of 10 kHz to 0.1 Hz with an AC amplitude of 10% DC current.

**9. Computational method.** The Vienna Ab initio Simulation Package (VASP) was used to perform all density functional theory (DFT) calculations. The generalized gradient approximation (GGA) in the revised Perdew-Burke-Ernzerhof (r-PBE) function have chosen the projected augmented wave potentials to describe the ionic cores and take valence electrons into account using a plane wave basis set with a kinetic energy cutoff of 400 eV. The DFT-D3 semiempirical correction method was employed to describe van der Waals interactions. Geometry optimizations were performed with the force convergency smaller than 0.05 eV/Å. Monkhorst-Pack k-points of 1×1×1 was applied for all the calculations. The Gibbs free energy change (∆G) of each elementary reaction step during ORR were calculated using the computational hydrogen electrode (CHE) model. In this model, the chemical potential is equal to the energy of half of the gas-phase H_2_ at 0 V vs. RHE. The electrode potential, U vs. RHE, is taken into consideration by adding –eU when an electron transforming step occurs. That is G (U) = – neU, where e is the elementary charge of an electron, n is the number of proton-electron pairs transferred, and U is the applied potential. The Gibbs free energy was calculated by the following equation:

ΔG = ΔE + ΔE_ZPE_ – TΔS + ΔG (U) (10)

Where the value of ΔE, ΔE_ZPE_ and ΔS denotes the changes of DFT energy, the zero-point energy and the entropy at 298.15 K, respectively. The equilibrium potential U for ORR was determined to be 0.46 V vs. CHE alkaline media and 1.23 V vs. CHE acidic media where the reactant and product are at the same energy level.

The thermodynamic feasibility of associative ORR on different active sites was investigated using a computational hydrogen electrode model. The 4e^-^ reaction steps for associative ORR in the alkaline medium are as follows ^[23]^:

O_2_(*g*) + 2H_2_O(*l*) + 4e^-^ + * → OOH* + OH^-^ + H_2_O(*l*) + 3e^-^ (11a)

OOH* + OH^-^ + H_2_O(*l*) + 3e^-^ → O* + 2OH^-^ + H_2_O(*l*) + 2e^-^ (11b)

O* + 2OH^-^ + H_2_O(*l*) + 2e^-^ → OH* + 3OH^-^ + e^-^  (11c)

O* + 3OH^-^ + e^-^ → 4OH^-^ + * (11d)

The 4e^-^ reaction steps for associative ORR in the acidic medium are as follows:

O_2_(*g*) + 4H^+^ + 4e^-^ + * → OOH* + 3H^+^ + 3e^-^ (12a)

OOH* + 3H^+^ + 3e^-^ → O* + H_2_O(*l*) + 2H^+^ + 2e^-^ (12b)

O* + H_2_O(*l*) + 2H^+^ + 2e^-^ → O* + H_2_O(*l*) + H^+^ + e^-^ (12c)

O* + H_2_O(*l*) + H^+^ + e^-^ → 2H_2_O(*l*) + * (12d)

where * represents the active sites. The ORR intermediates (OOH*, O*, and OH*) were optimized on different active sites using DFT calculations.

**10. Statistical Analysis.** All statistical analyses were performed to ensure the reliability and reproducibility of the electrocatalytic performance data and structural characterization results of Fe/N-doped carbon model catalysts. The following protocols were strictly followed:

**(1) Data Pre-processing.** Raw data (electrochemical performance, structural characterization parameters) were pre-processed. No data transformation or normalization was performed, as all measurements were obtained under consistent conditions.

**(2) Data Presentation.** The quantitative data of in-situ nitrite stripping experiments in the manuscript and supporting information are presented as mean±standard deviation (SD).

**(3) Sample Size.** The sample size (n) for each analysis was: Electrochemical tests (ORR activity, nitrite stripping): n = 5; Physical/chemical characterization (XRD, Raman, XPS, BET): n = 3; DFT calculations: n = 1 (no replication required for converged theoretical results).

**(4)** **Statistical Methods for Significant Differences**. Two-tailed Student’s t-test was used for pairwise comparisons, and one-way ANOVA for multi-group comparisons. Key details: Testing type: Two-sided (no a priori directional hypothesis). Significance level: α = 0.05; P < 0.05 was considered significant, P < 0.01 highly significant. Post-hoc test: Tukey’s HSD test was applied for significant ANOVA results (no alpha adjustment). Assumptions validity: Normality (Shapiro-Wilk test) and homogeneity of variance (Levene’s test) were verified; all pre-processed data satisfied these assumptions.

**(5) Software Used.** The particle size distribution of the catalyst was statistically analyzed using the Nano Measurer 1.2. Data visualization and raw data organization were conducted via OriginPro 2021.

**Supplementary Figures**


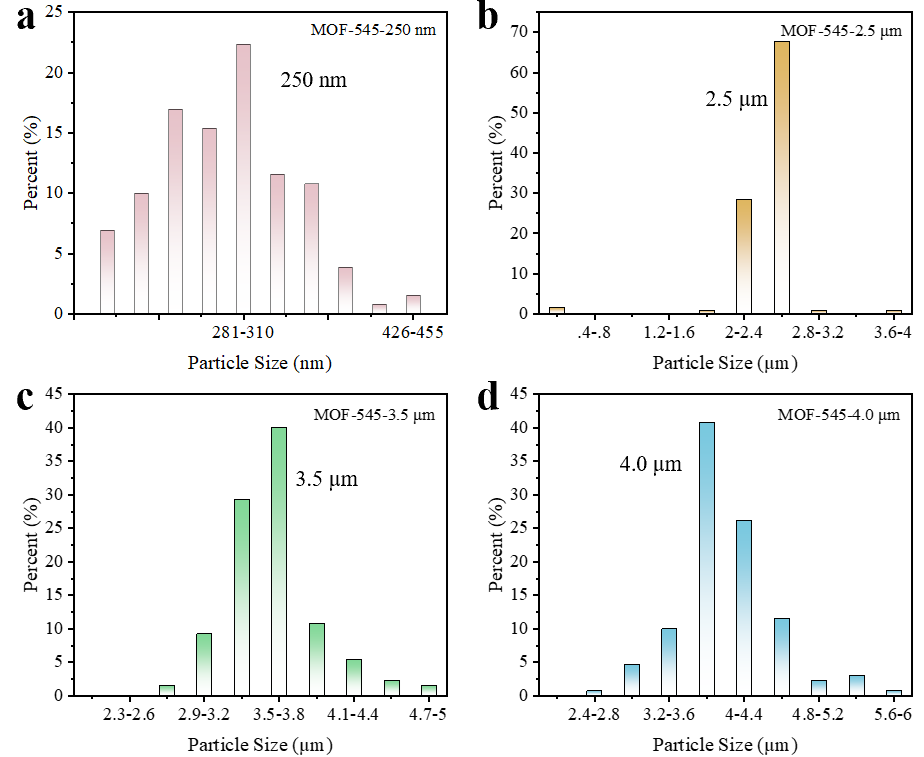


**Fig. S1.** (a-d) The sectional size distribution of the rod-like MOF-545-*x*.


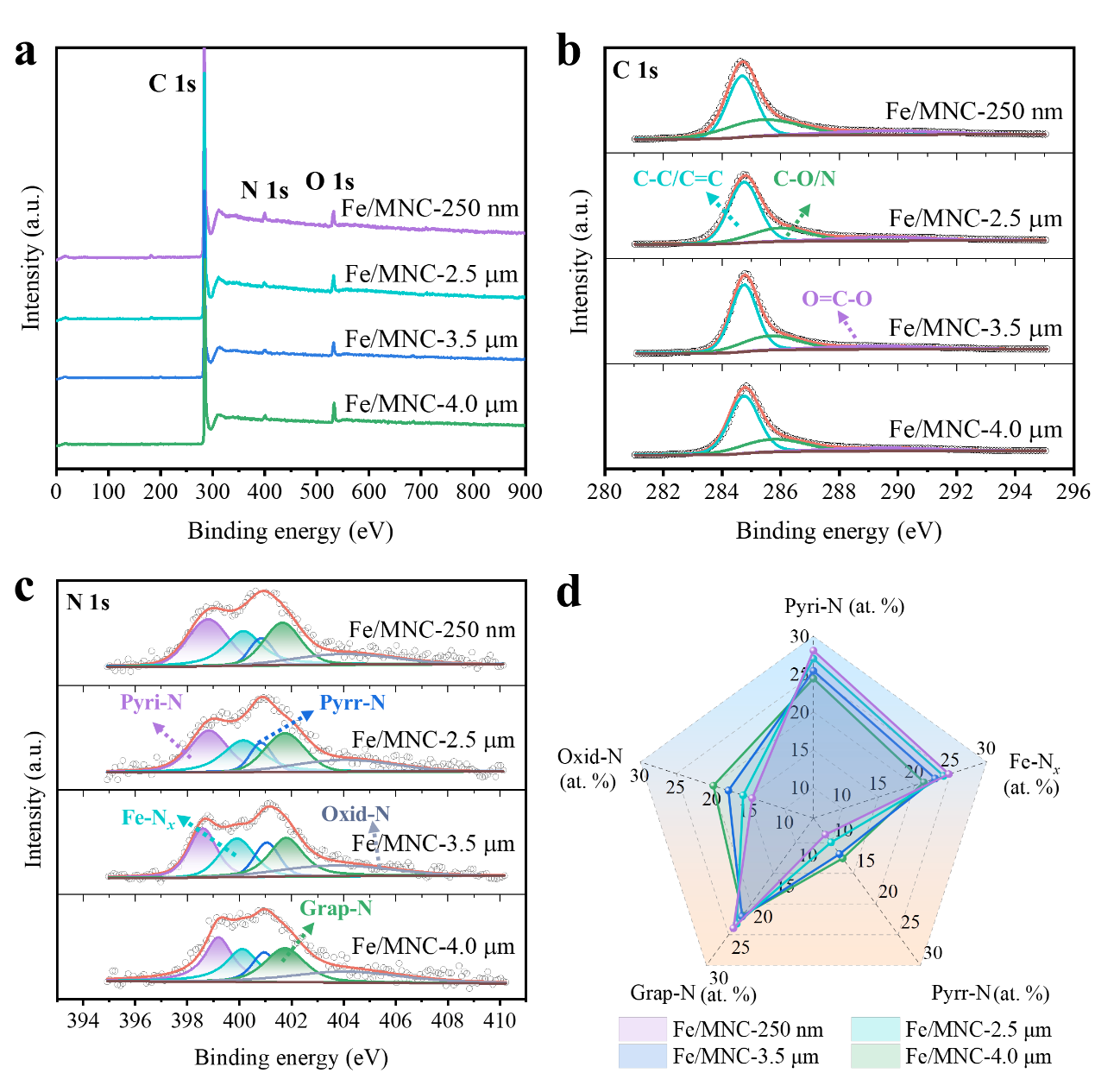


**Fig. S2.** (a) The wide-range XPS spectra and (b) C1s spectra of Fe/MNC-*x*.**Table S1.** The specific surface area (S_BET_), pore volume (V_pore_) and pore size (S_pore_) of the samples

| **Sample** | **S_BET_ (m^2^ g^-1^)** | **S_pore_ (nm)** | **V_pore_ (cm^3^ g^-1^)** |
| --- | --- | --- | --- |
| Fe/MNC-250 nm | 639.61 | 12.41 | 1.958 |
| Fe/MNC-2.5 μm | 675.72 | 8.322 | 1.219 |
| Fe/MNC-3.5 μm | 631.91 | 8.428 | 1.242 |
| Fe/MNC-4.0 μm | 603.33 | 8.089 | 1.159 |

**Table S2.** The elemental content (at.%) in Fe/MNC-*x*

| **Sample** | **C (at.%)** | **N (at.%)** | **O (at.%)** | **Fe (at.%)** |
| --- | --- | --- | --- | --- |
| Fe/MNC-250 nm | 93.05 | 0.41 | 2.96 | 3.58 |
| Fe/MNC-2.5 μm | 92.82 | 0.32 | 3.35 | 3.51 |
| Fe/MNC-3.5 μm | 92.69 | 0.41 | 2.91 | 3.99 |
| Fe/MNC-4.0 μm | 93.3 | 0.32 | 2.43 | 3.94 |

**Table S3.** The types of nitrogen and their percentages doped in Fe/MNC-*x*

| **Sample** | **Pyridinic N**  **(at.%)** | **Pyrrolic N**  **(at.%)** | **Graphitic N (at.%)** | **Oxidized N**  **(at.%)** | **Fe-N*_x_***  **(at.%)** |
| --- | --- | --- | --- | --- | --- |
| Fe/MNC-250 nm | 28.09 | 8.69 | 23.95 | 14.49 | 24.78 |
| Fe/MNC-2.5 μm | 27.03 | 9.95 | 23.22 | 15.75 | 24.05 |
| Fe/MNC-3.5 μm | 25.42 | 11.86 | 22.04 | 17.80 | 22.88 |
| Fe/MNC-4.0 μm | 24.40 | 12.55 | 21.87 | 19.87 | 21.31 |


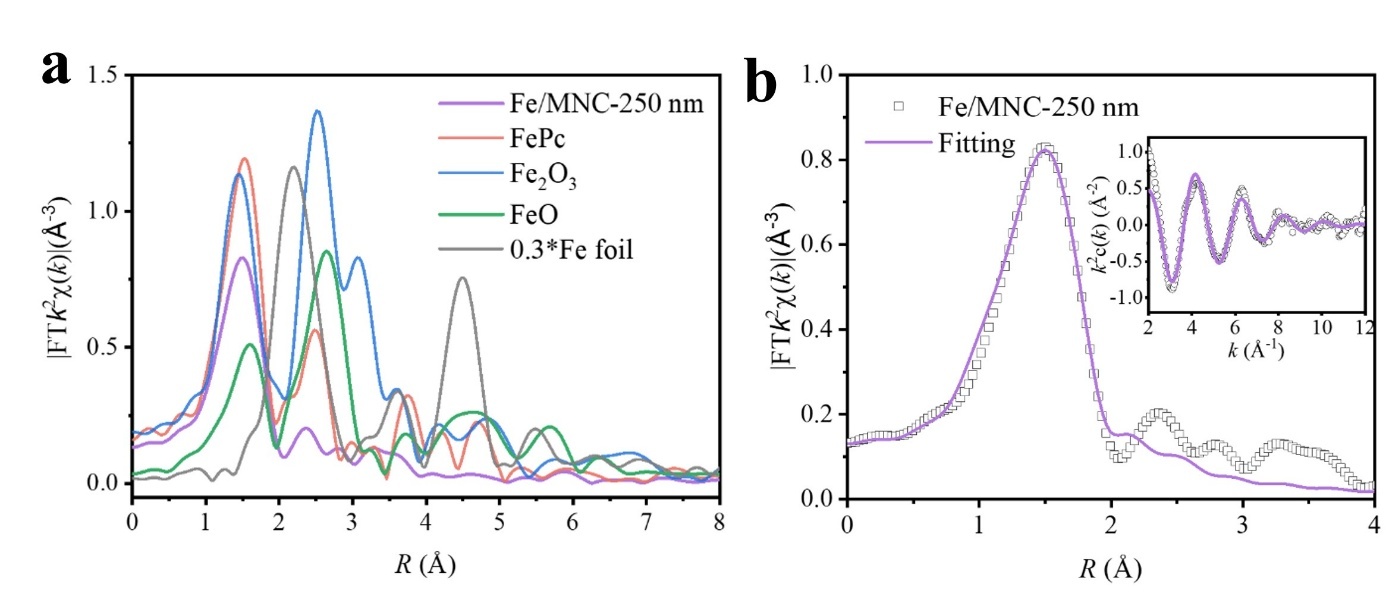


**Fig. S3** (a) FT-EXAFS spectra of the Fe/MNC-250 nm catalyst and reference samples (FeO, Fe_2_O_3_, FePc, and standard Fe foil). (b) Fourier transform Fe K-edge EXAFS spectrum (open circles) and fitting curve (fuchsia line) for the Fe/MNC-250 nm specimen, inset shows the EXAFS k space fitting curves.

**Table S4.** EXAFS fitting parameters of Ru *K*-edge for sample.

| Sample | Shell | N ^a)^ | R ^b)^ [Å] | σ^2^ ^c)^ [Å^2^] | R factor ^d)^ [%] |
| --- | --- | --- | --- | --- | --- |
| Fe/MNC-*x* | N | 4.0±0.3 | 1.98 | 0.010 | 0.04 |
|  | O | 1.0±0.2 | 2.09 | 0.005 | - |

^a)^ CN: Coordination numbers; ^b)^ R: Bond distance; ^c)^ σ^2^: Debye-Waller factors; ^d)^ R factor: The values of R factor quantify the goodness of fit when fitting all data scans for sample.


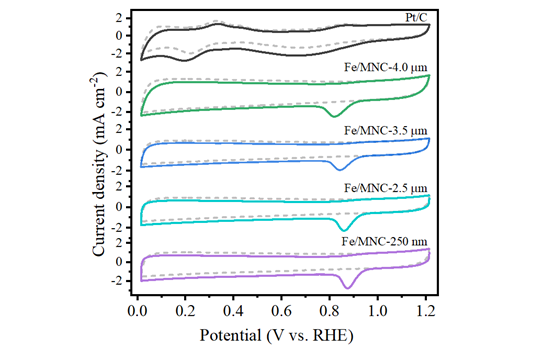


**Fig. S4.** CV curves for the Fe/MNC-*x* and Pt/C under O_2_-saturated (solid lines) or N_2_-saturated (dashed lines) conditions.


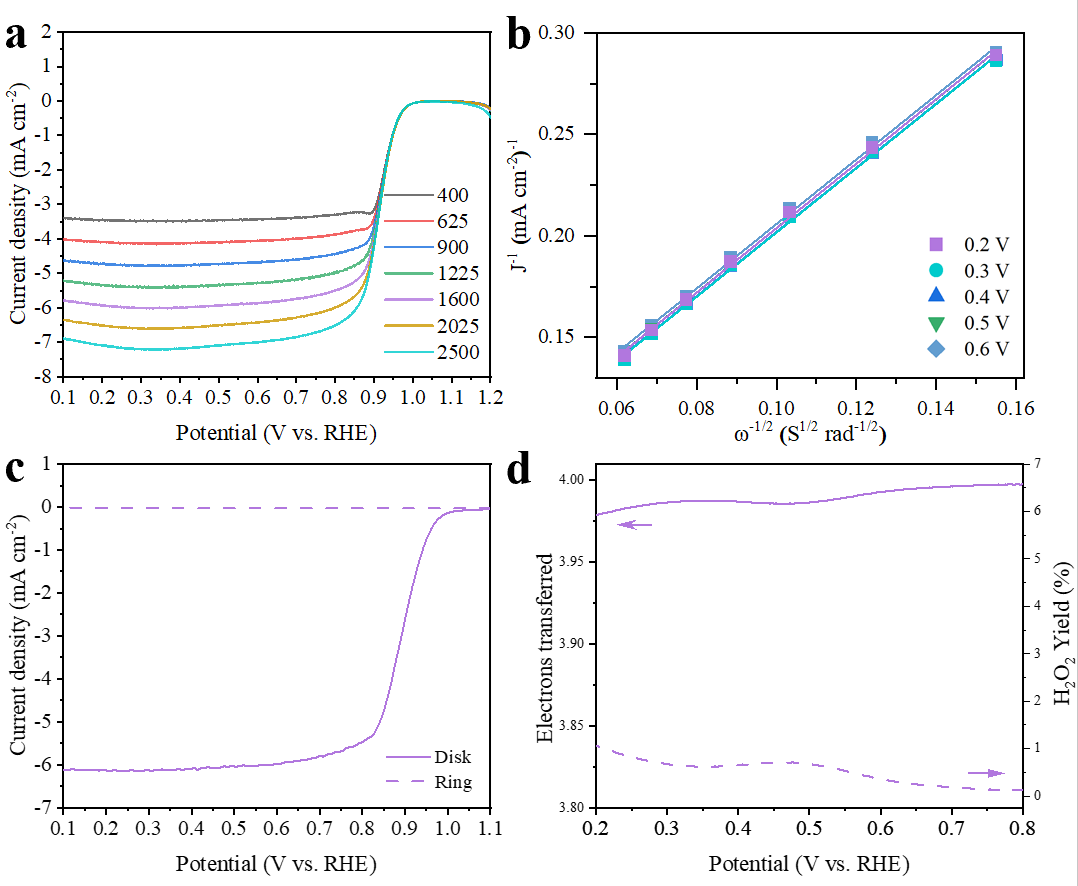


**Fig. S5.** (a) LSV of the Fe/MNC-250 nm electrode with different rotation rates (400-2500 rpm) in O_2_ saturated 0.1 M KOH. (b) The corresponding K-L plots at different potentials. (c) RRDE curve of Fe/MNC-250 nm with a scan rate of 10 mV s^-1^. (d) Electron transfer number and peroxide yield of Fe/MNC-250 nm.


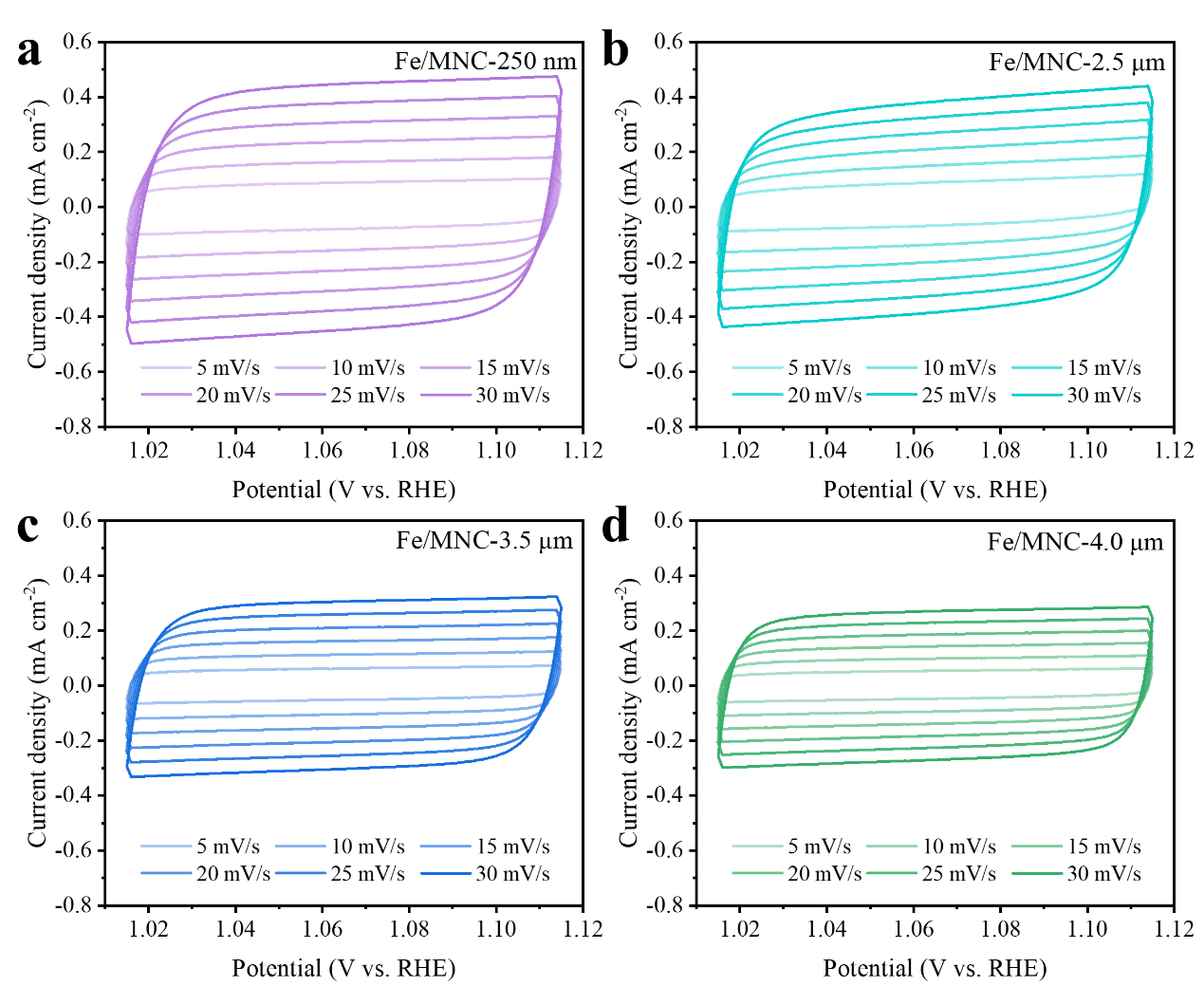


**Fig. S6.** (a-d) CV curves for Fe/MNC-*x* at various scan rates (5-30 mV s^−1^).


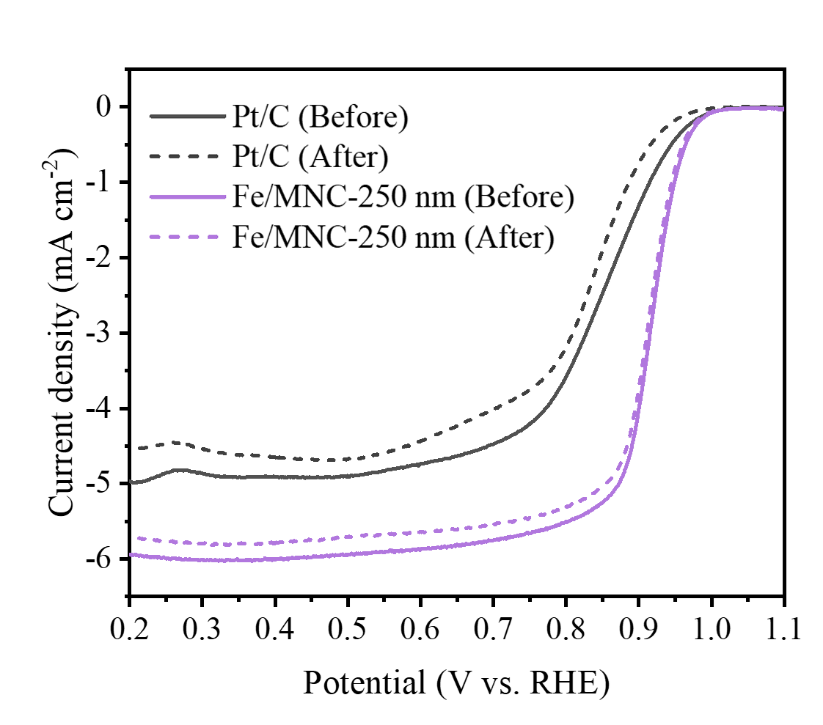


**Fig. S7.** Comparison of LSV curves for Pt/C and Fe/MNC-250 nm before and after 5000 continuous CV cycles with a scan rate of 10 mV s^-1^ at 1600 rpm.

**Table S5.** Comparison of the ORR performance of Fe/MNC-*x* with other recently reported carbon-based catalysts in O_2_ saturated 0.1 M KOH. (Note: E_onset_: on-set potential; E_1/2_: half-wave potential; J_L_: diffusion limiting current density; n: electron transfer number.)

| **Catalyst** | **E_onset_**  **(V vs. RHE)** | **E_1/2_**  **(V vs. RHE)** | **J_L_**  **(mA·cm^-2^)** | **n** | **Loading amount (mg·cm^-2^)** | **Ref.** |
| --- | --- | --- | --- | --- | --- | --- |
| 20 wt.% Pt/C | 0.98 | 0.851 | 4.88 | 3.972 | 0.20 | This  work |
| Fe/MNC-250 nm | 1.028 | 0.917 | 6.06 | 3.997 | 0.20 |  |
| Fe/MNC-2.5 μm | 0.987 | 0.875 | 5.92 | 3.992 | 0.20 |  |
| Fe/MNC-3.5 μm | 0.965 | 0.855 | 5.34 | 3.987 | 0.20 |  |
| Fe/MNC-4.5 μm | 0.955 | 8.836 | 4.99 | 3.970 | 0.20 |  |
| Co‐HTA‐CN | 0.90 | 0.800 | 0.79 | 3.70 | 0.64 | [2] |
| Co@*rhm*-PorBTD | 0.89 | 0.830 | 0.27 | - | 0.15 | [3] |
| 2.0 wt% Cu-N-C | 0.88 | 0.830 | 5.4 | 3.91(9) | 0.20 | [4] |
| CAN-Pc(Co)-*p* | 1.05 | 0.850 | 5.5 | 3.84 | 0.02 | [5] |
| Zn/CoN-C | 1.004 | 0.861 | 6.1 | 3.88 | 0.255 | [6] |
| o-MQFe-10:20:5 | 0.96 | 0.861 | 5.5 | 4.0 | 0.65 | [7] |
| FeNSC-ZM | 1.06 | 0.870 | 9.8 | 4.0 | 0.50 | [8] |
| (Fe,Co/DSA-NSC) | - | 0.879 | 5.3 | 3.9 | 0.25 | [9] |
| FeNCS | - | 0.882 | 5.6 | 3.93 | 0.50 | [10] |
| Fe-CNG | 0.96 | 0.890 | 6.0 | 4.03 | 0.20 | [11] |
| CoNC-0.5C_6_H_14_O_6_-800 | 1.01 | 0.890 | 5.5 | 4.0 | 0.31 | [12] |
| FeM_nac_/Mn-N_4_C | 1.00 | 0.900 | 5.9 | 3.97 | 0.80 | [13] |
| FeAB–O | - | 0.900 | 6.1 | 3.96 | 0.20 | [14] |
| Fe/Zn–N–C | 1.01 | 0.906 | 7 | 4.02 | 0.38 | [15] |
| 1100HCS-O-FePc | 0.98 | 0.910 | 5.7 | 3.90 | 0.06 | [16] |
| Fe-SAs@NCTCs | 1.02 | 0.910 | 5.4 | 4.0 | 0.255 | [17] |
| Mg–N–C | 1.03 | 0.910 | 5.2 | 4.0 | 0.20 | [18] |
| Fe_2_S_2_@CN | 1.07 | 0.920 | 5.6 | 4.0 | - | [19] |
| FeNC-Ce-950 | - | 0.921 | 5.3 | 4.0 | 0.80 | [20] |
| Fe/Mn-N-C | - | 0.926 | 4.6 | - | 0.60 | [21] |
| FePc-*β*-NO_2_-KJ | 1.0 | 0.930 | 6.0 | 4.0 | 0.60 | [22] |


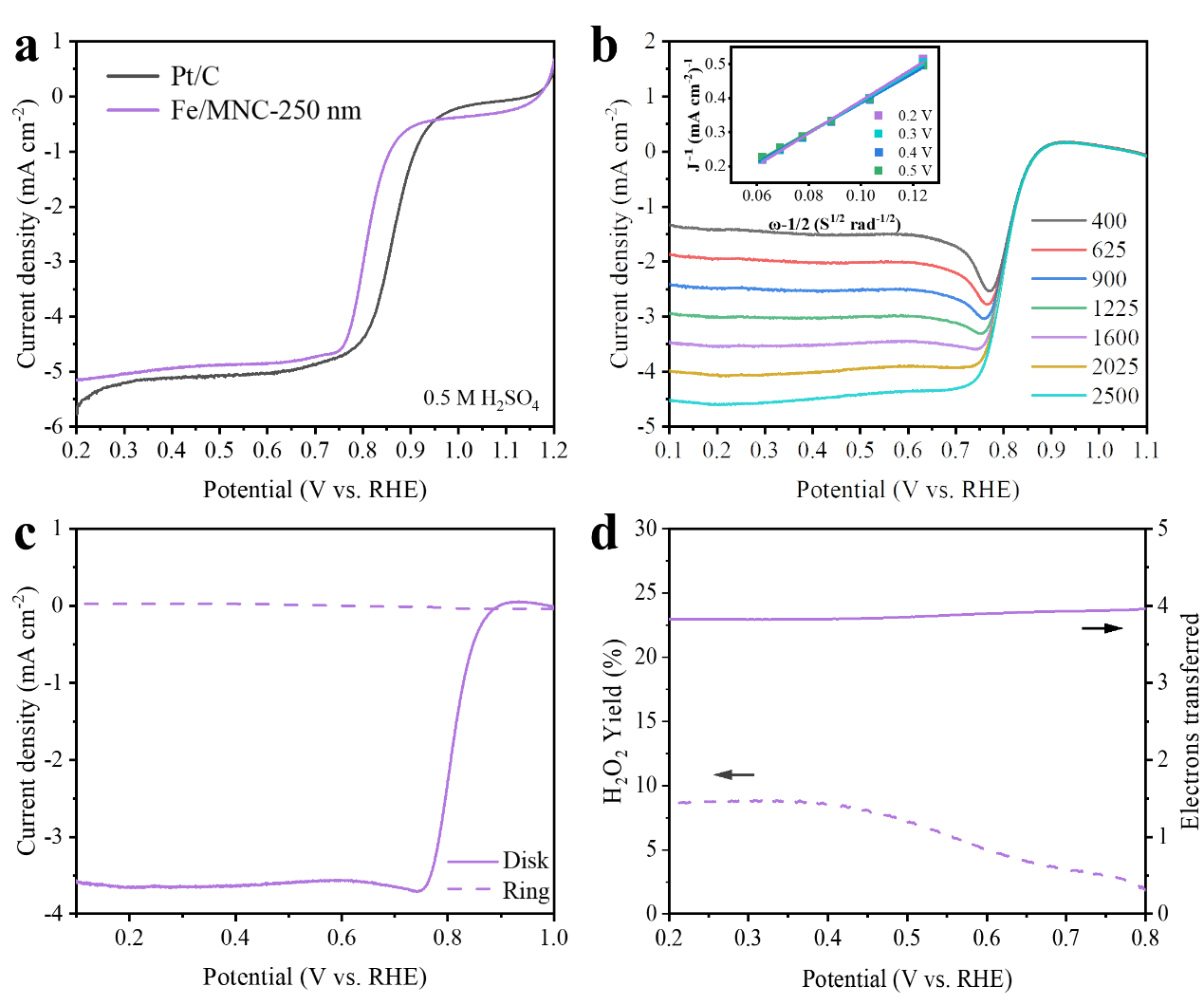


**Fig. S8.** ORR performances of Fe/MNC-250 nm in O_2_ saturated 0.5 M H_2_SO_4_. (a) LSV curves of Pt/C and Fe/MNC-250 nm. (b) LSV curves with different rotation rates, the corresponding K-L plots at different potentials. (c) RRDE curve. (d) Electron transfer number and peroxide yield.

**Note for Fig. S8:** LSV curves at different rotating rates of rotating disk electrodes were recorded (**Fig. S8a**). The electron transfer number during ORR has been estimated using the K-L plots (**Fig. S8b**), and the value around 4. The electron transfer number also has been estimated using the rotating ring-disk electrode LSV curves (**Fig. S8c and d**), which provide values 3.83 to 3.96, and the yield of H_2_O_2_ is 8.6% to 0, respectively, implying that the Fe/MNC-250 nm catalyst proceeds via the four-electron dominant ORR pathway.


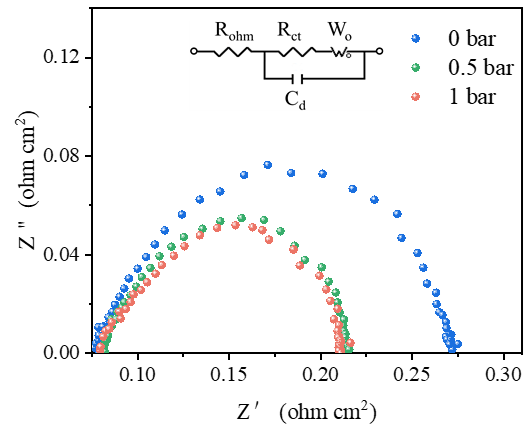


**Fig. S9.** Nyquist plots for PEMFCs of cathode catalysts at the current density of 1.5 A cm^-2^, the inset shows the equivalent circuit model. Z′ and Z″ are the real and imaginary parts of the impedance.


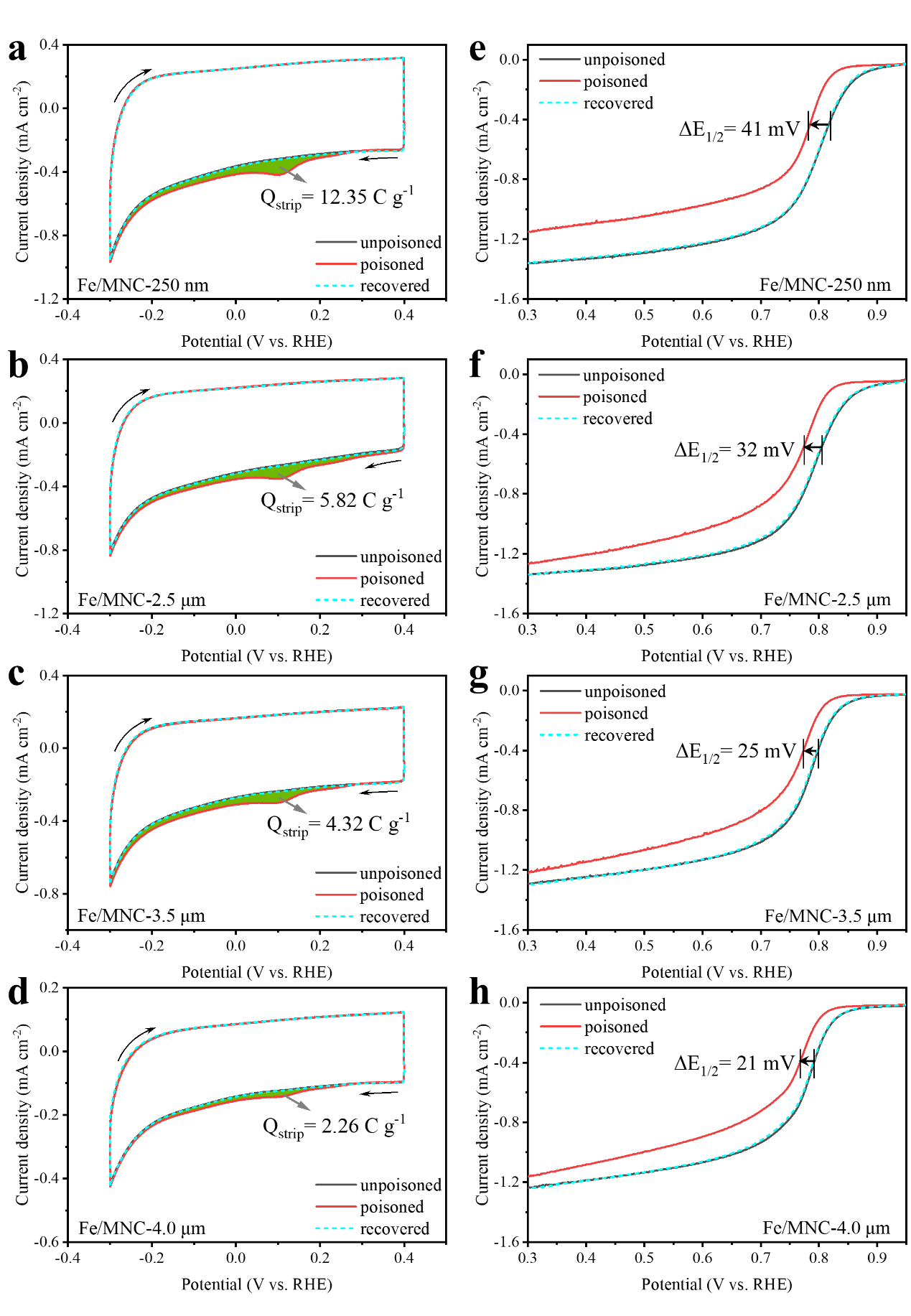


**Fig. S10.** (a-d) CV in the nitrite reductive nitrite stripping region before, during and after nitrite adsorption. (e-h) LSV curves of Fe/MNC-*x* before, during and after nitrite adsorption.


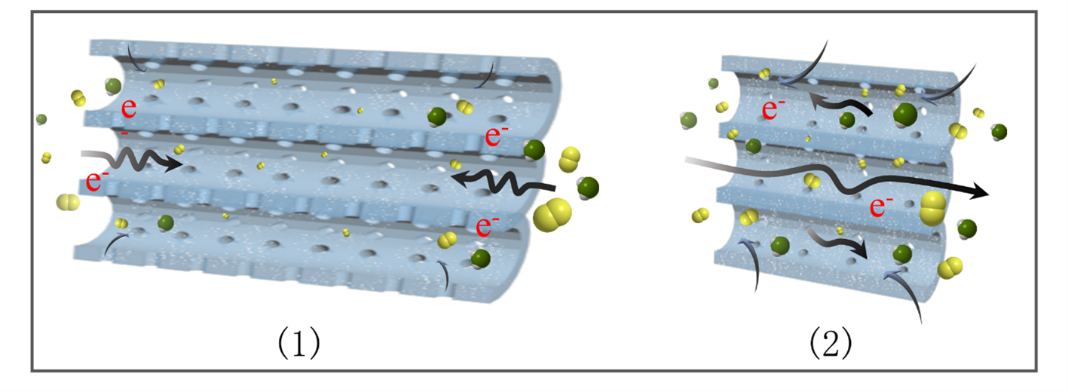


**Fig. S11.** Schematic illustration of the mass transport of tunable size Fe/MNC-*x*.


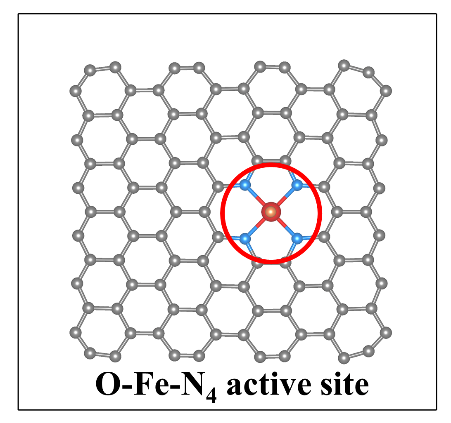


**Fig. S12.** The theoretical structure model of O-Fe-N_4_.


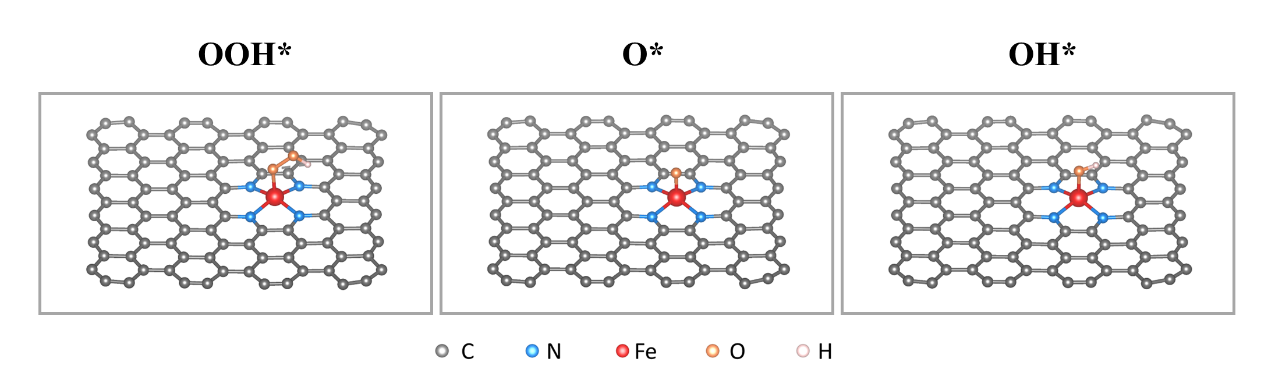


**Fig. S13.** The optimized atomic configurations of adsorbed intermediates states (OOH*, O*, and OH*) on O-Fe-N_4_ active site, corresponding to the free energy diagram in **Table. S6 and 7**.

**Table S6.** The adopted values of entropy and zero-point energy for the adsorption and gas phase states of molecules.

|  | H_2_O | H_2_ | O_2_ | OOH* | O* | OH* |
| --- | --- | --- | --- | --- | --- | --- |
| ZPE (eV) | 0.58 | 0.34 | 0.10 | 0.40 | 0.07 | 0.32 |
| TS (eV) | 0.67 | 0.40 | 0.64 | 0.14 | 0.06 | 0.08 |

**Table S7.** Free energy changes at U = 0 V and U = 0.46 V for the 4-electron (4e^-^) pathways on O-Fe-N_4_ active site during the ORR in the alkaline medium

| 4e^-^ reaction steps in alkaline medium | | **ΔG (eV)** |
| --- | --- | --- |
| **U = 0 V** | step 11a | 0.28 |
|  | step 11b | -1.76 |
|  | step 11c | 0.05 |
|  | step 11d | -0.41 |
| **U = 0.46 V** | step 11a | 0.74 |
|  | step 11b | -1.3 |
|  | step 11c | 0.51 |
|  | step 11d | 0.05 |

**Table S8.** Free energy changes at U = 0 V and U = 1.23 V for the 4-electron (4e^-^) pathways on O-Fe-N_4_ active site during the ORR in the acidic medium

| 4e^-^ reaction steps in acidic medium | | **ΔG (eV)** |
| --- | --- | --- |
| **U = 0 V** | step 12a | -0.55 |
|  | step 12b | -2.59 |
|  | step 12c | -0.78 |
|  | step 12d | -1.00 |
| **U = 1.23 V** | step 12a | 0.68 |
|  | step 12b | -1.36 |
|  | step 12c | 0.45 |
|  | step 12d | 0.23 |

**Supplementary References**

[1] Dawei Feng, Zhi-Yuan Gu, Jian-Rong Li, Hai-Long Jiang, Zhangwen Wei, and Hong-Cai Zhou. Zirconium-metalloporphyrin PCN-222: Mesoporous metal-organic frameworks with ultrahigh stability as biomimetic catalysts. Angewandte Chemie International Edition. 2012, 41, 10453-10456.

[2] Sijia Liu, Minghao Liu, Xuewen Li, Shuai Yang, Qiyang Miao, Qing Xu, Gaofeng Zeng. Metal organic polymers with dual catalytic sites for oxygen reduction and oxygen evolution reactions. Carbon Energy, 2023, 5(5), 1-11.

[3] Subhajit Bhunia, Armando Peña-Duarte, Huifang Li, Hong Li, Mohamed Fathi Sanad, Pranay Saha, Matthew A. Addicoat, Kotaro Sasaki, T. Amanda Strom, Miguel José Yacamán, Carlos R. Cabrera, Ram Seshadri, Santanu Bhattacharya, Jean-Luc Brédas, and Luis Echegoyen. [2, 1, 3]-Benzothiadiazole-spaced co-porphyrin-based covalent organic frameworks for O_2_ reduction. ACS nano, 2023, 17(4): 3492-3505.

[4] Ji Yang, Wengang Liu, Mingquan Xu, Xiaoyan Liu, Haifeng Qi, Leilei Zhang, Xiaofeng Yang, Shanshan Niu, Dan Zhou, Yuefeng Liu, Yang Su, Jian-Feng Li, Zhong-Qun Tian, Wu Zhou, Aiqin Wang, and Tao Zhang. Dynamic behavior of single-atom catalysts in electrocatalysis: Identification of Cu-N_3_ as an active site for the oxygen reduction reaction. Journal of the American Chemical Society, 2021, 143(36): 14530-14539.

[5] Shaoxuan Yang, Yihuan Yu, Meiling Dou, Zhengping Zhang, and Feng Wang. Edge-functionalized polyphthalocyanine networks with high oxygen reduction reaction activity. Journal of the American Chemical Society, 2020, 142(41): 17524-17530.

[6] Ziyang Lu, Bo Wang, Yongfeng Hu, Wei Liu, Yufeng Zhao, Ruoou Yang, Zhiping Li, Jun Luo, Bin Chi, Zheng Jiang, Minsi Li, Shichun Mu, Shijun Liao, Jiujun Zhang, and Xueliang Sun. An isolated Zinc–Cobalt atomic pair for highly active and durable oxygen reduction. Angewandte Chemie, 2019, 131(9): 2648-2652.

[7] Yarong Liu, Xiangjian Liu, Zunhang Lv, Rui Liu, Liuhua Li, Jinming Wang, Wenxiu Yang, Xin Jiang, Xiao Feng, and Bo Wang. Tuning the spin state of the iron center by bridge-bonded Fe‐O‐Ti ligands for enhanced oxygen reduction. Angewandte Chemie, 2022, 134(21): e202117617.

[8] Yaqian Dong, Zhi Fang, Deliu Ou, Qing Shi, Yu Ma, Weiyou Yang, Bin Tang, Qiao Liu. Rational fabrication of S-modified Fe–N–C nanosheet electrocatalysts for efficient and stable pH-universal oxygen reduction. Chemical Engineering Journal, 2022, 444: 136433.

[9] Ghulam Yasin, Sajjad Ali, Shumaila Ibraheem, Anuj Kumar, Mohammad Tabish, Muhammad Asim Mushtaq, Saira Ajmal, Muhammad Arif, Muhammad Abubaker Khan, Ali Saad, Liang Qiao, and Wei Zhao. Simultaneously engineering the synergistic-effects and coordination-environment of dual-single-atomic Iron/Cobalt-sites as a bifunctional oxygen electrocatalyst for rechargeable Zinc-Air batteries. ACS Catalysis, 2023, 13(4): 2313-2325.

[10] Feng Li, Gao-Feng Han, Yunfei Bu, Hyuk-Jun Noh, Jong-Pil Jeon, Tae Joo Shin, Seok-Jin Kim, Yuen Wu, Hu Young Jeong, Zhengping Fu, Yalin Lu, and Jong-Beom Baek. Revealing isolated M−N_3_C_1_ active sites for efficient collaborative oxygen reduction catalysis. Angewandte Chemie International Edition, 2020, 59(52): 23678-23683.

[11] Wenchao Wan, Yonggui Zhao, Shiqian Wei, Carlos A. Triana, Jingguo Li, Andrea Arcifa, Christopher S. Allen, Rui Cao, and Greta R. Patzke. Mechanistic insight into the active centers of single/dual-atom Ni/Fe-based oxygen electrocatalysts. Nature Communications, 2021, 12(1): 5589.

[12] Shaojie Shi, Biaolong Wang, Yifei Wang, Yanmin Yang, Zhiguo Zhang, Yousheng Xu, Yange Suo. Structure optimization of ZIF-12-derived Co-NC for efficient oxygen reduction and oxygen evolution. Fuel, 2022, 330: 125516.

[13] Heng Liu, Luozhen Jiang, Javid Khan, Xinxin Wang, Jiamin Xiao, Handong Zhang, Haijiao Xie, Lina Li, Shuangyin Wang, and Lei Han. Decorating single-atomic Mn sites with FeMn clusters to boost oxygen reduction reaction. Angewandte Chemie, 2023, 135(3): e202214988.

[14] Kejun Chen, Kang Liu, Pengda An, Huangjingwei Li, Yiyang Lin, Junhua Hu, Chuankun Jia, Junwei Fu, Hongmei Li, Hui Liu, Zhang Lin, Wenzhang Li, Jiahang Li, Ying-Rui Lu, Ting-Shan Chan, Ning Zhang and Min Liu. Iron phthalocyanine with coordination induced electronic localization to boost oxygen reduction reaction. Nature Communications, 2020, 11(1): 4173.

[15] Hongguan Li, Shuanlong Di, Ping Niu, Shulan Wang, Jing Wang, and Li Li. A durable half-metallic diatomic catalyst for efficient oxygen reduction. Energy & Environmental Science, 2022, 15(4): 1601-1610.

[16] Xuhui Li, Ruixue Zhao, Yuanyuan Fu, Dawei Xu, Yunpeng Kang, Kai Li, Zhongfeng Li, Lirong Zheng, Xia Zuo. N/O-co-doped carbon shell structures loaded with iron phthalocyanine for oxygen reduction catalysis. ChemCatChem, 2022, 14(18): e202200517.

[17] Fenghong Lu, Kaicai Fan, Lixiu Cui, Bin Li, Yu Yang, Lingbo Zong, Lei Wang. Engineering FeN_4_ active sites onto nitrogen-rich carbon with tubular channels for enhanced oxygen reduction reaction performance. Applied Catalysis B: Environmental, 2022, 313: 121464.

[18] Shuai Liu, Zedong Li, Changlai Wang, Weiwei Tao, Minxue Huang, Ming Zuo, Yang Yang, Kang Yang, Lijuan Zhang, Shi Chen, Pengping Xu, Qianwang Chen. Turning main-group element magnesium into a highly active electrocatalyst for oxygen reduction reaction. Nature Communications, 2020, 11(1): 938.

[19] Ming Wang, Zhong Zhang, Songlin Zhang, Wei Liu, Wenzhe Shang, Xiaofang Su, Yan Liang, Furi Wang, Xujiao Ma, Yiwei Liu, and Yadong Li. Non‐planar nest‐like [Fe_2_S_2_] cluster sites for efficient oxygen reduction catalysis. Angewandte Chemie, 2023, 62, e202300826.

[20] Feng-Di Tu, Zi-Yun Wu, Pan Guo, Li-Xiao Shen, Zi-Yu Zhang, Yun-Kun Dai, Miao Ma, Jing Liu, Bin Xu, Yun-Long Zhang, Lei Zhao, Zhen-Bo Wang. Fe-NC Catalysts decorated with oxygen vacancies-rich CeO_x_ to increase oxygen reduction performance for Zn-air batteries. Journal of Colloid and Interface Science, 2023, 637: 10–19.

[21] Xuan Luo, Wenkun Wu, Youheng Wang, Yuyang Li, Jinyu Ye, Haoyu Wang, Qiaorong Jiang, Zhiyou Zhou, Yuguang C. Li, Yucheng Wang, and Shigang Sun. Relay catalysis of multi-sites promotes oxygen reduction reaction. Advanced Functional Materials, 2023: 2215021.

[22] Yang Wang, Tianpei Zhou, Shanshan Ruan, Hu Feng, Wentuan Bi, Jun Hu, Ting Chen, Hongfei Liu, Bingkai Yuan, Nan Zhang, Wenjie Wang, Lidong Zhang, Wangsheng Chu, Changzheng Wu, and Yi Xie. Directional manipulation of electron transfer by energy level engineering for efficient cathodic oxygen reduction. Nano Letters, 2022, 22(16): 6622-6630.

[23] Qing Lv, Wenyan Si, Jianjiang He, Lei Sun, Chunfang Zhang, Ning Wang, Ze Yang, Xiaodong Li, Xin Wang, Weiqiao Deng, Yunze Long, Changshui Huang and Yuliang Li. Selectively nitrogen-doped carbon materials as superior metal-free catalysts for oxygen reduction. Nat. Commun. 2018, 9, 3376.
